# A COVID-19 vaccine candidate using SpyCatcher multimerization of the SARS-CoV-2 spike protein receptor-binding domain induces potent neutralising antibody responses

**DOI:** 10.1038/s41467-020-20654-7

**Published:** 2021-01-22

**Authors:** Tiong Kit Tan, Pramila Rijal, Rolle Rahikainen, Anthony H. Keeble, Lisa Schimanski, Saira Hussain, Ruth Harvey, Jack W. P. Hayes, Jane C. Edwards, Rebecca K. McLean, Veronica Martini, Miriam Pedrera, Nazia Thakur, Carina Conceicao, Isabelle Dietrich, Holly Shelton, Anna Ludi, Ginette Wilsden, Clare Browning, Adrian K. Zagrajek, Dagmara Bialy, Sushant Bhat, Phoebe Stevenson-Leggett, Philippa Hollinghurst, Matthew Tully, Katy Moffat, Chris Chiu, Ryan Waters, Ashley Gray, Mehreen Azhar, Valerie Mioulet, Joseph Newman, Amin S. Asfor, Alison Burman, Sylvia Crossley, John A. Hammond, Elma Tchilian, Bryan Charleston, Dalan Bailey, Tobias J. Tuthill, Simon P. Graham, Helen M. E. Duyvesteyn, Tomas Malinauskas, Jiandong Huo, Julia A. Tree, Karen R. Buttigieg, Raymond J. Owens, Miles W. Carroll, Rodney S. Daniels, John W. McCauley, David I. Stuart, Kuan-Ying A. Huang, Mark Howarth, Alain R. Townsend

**Affiliations:** 1 MRC Human Immunology Unit, MRC Weatherall Institute of Molecular Medicine, John Radcliffe Hospital, University of Oxford, Oxford, OX3 9DS UK; 2 Centre for Translational Immunology, Chinese Academy of Medical Sciences Oxford Institute, University of Oxford, Oxford, UK; 3 Department of Biochemistry, University of Oxford, South Parks Road, Oxford, OX1 3QU UK; 4 Worldwide Influenza Centre, The Francis Crick Institute, 1 Midland Road, London, NW1 1AT UK; 5 The Pirbright Institute, Ash Road, Pirbright, GU24 0NF UK; 6 Department of Microbial Sciences, Faculty of Health and Medical Sciences, University of Surrey, Guildford, GU2 7XH UK; 7 Division of Structural Biology, Nuffield Department of Medicine, University of Oxford, The Wellcome Centre for Human Genetics, Headington, Oxford UK; 8 Rutherford Appleton Laboratory, Protein Production UK, Research Complex at Harwell, and Rosalind Franklin Institute, Harwell, Didcot OX11 0FA UK; 9 National Infection Service, Public Health England, Porton Down, Salisbury SP4 0JG UK; 10 Nuffield Department of Medicine, Wellcome Trust Centre for Human Genetics, University of Oxford, Oxford, OX3 7BN UK; 11 Diamond Light Source Ltd, Harwell Science & Innovation Campus, Didcot, UK; 12 Research Center for Emerging Viral Infections, College of Medicine, Chang Gung University, Taoyuan, Taiwan; 13 Division of Pediatric Infectious Diseases, Department of Pediatrics, Chang Gung Memorial Hospital, Taoyuan, Taiwan

**Keywords:** Viral infection, Protein vaccines, SARS-CoV-2, Preclinical research

## Abstract

There is need for effective and affordable vaccines against SARS-CoV-2 to tackle the ongoing pandemic. In this study, we describe a protein nanoparticle vaccine against SARS-CoV-2. The vaccine is based on the display of coronavirus spike glycoprotein receptor-binding domain (RBD) on a synthetic virus-like particle (VLP) platform, SpyCatcher003-mi3, using SpyTag/SpyCatcher technology. Low doses of RBD-SpyVLP in a prime-boost regimen induce a strong neutralising antibody response in mice and pigs that is superior to convalescent human sera. We evaluate antibody quality using ACE2 blocking and neutralisation of cell infection by pseudovirus or wild-type SARS-CoV-2. Using competition assays with a monoclonal antibody panel, we show that RBD-SpyVLP induces a polyclonal antibody response that recognises key epitopes on the RBD, reducing the likelihood of selecting neutralisation-escape mutants. Moreover, RBD-SpyVLP is thermostable and can be lyophilised without losing immunogenicity, to facilitate global distribution and reduce cold-chain dependence. The data suggests that RBD-SpyVLP provides strong potential to address clinical and logistic challenges of the COVID-19 pandemic.

## Abstract (web summary)

Vaccines for SARS-COV-2 are needed in the ongoing pandemic. Here the authors characterize a vaccine candidate that presents the receptor-binding domain (RBD) of SARS-CoV-2 spike protein on a synthetic VLP platform using SpyTag/SpyCatcher technology and show immunogenicity of a prime-boost regimen in mice and pigs.

## Introduction

Coronavirus disease 2019 (COVID-19), caused by a novel coronavirus named severe acute respiratory syndrome coronavirus 2 (SARS-CoV-2), was first reported in Wuhan, China in December 2019^1^. Since then COVID-19 has spread across the world and was declared a pandemic by the World Health Organisation (WHO) in March 2020. As of August 2020, there have been over 20 million confirmed COVID-19 cases worldwide and around 800,000 deaths^2^. There are no vaccines or effective treatments for COVID-19 to date; however, as of August 2020, there are 48 vaccine candidates in clinical evaluation and around 160 are in pre-clinical testing^3^. Vaccine candidates in current clinical evaluation include inactivated, viral vector (replicating and non-replicating), protein subunit, nucleic acid (DNA and RNA) and virus-like particle (VLP) vaccines with the majority of them focusing on using the full-length SARS-CoV-2 spike glycoprotein (S) as an immunogen.

SARS-CoV-2 is an enveloped virus carrying a single-stranded positive-sense RNA genome (~30 kb), belonging to the genus *Betacoronavirus* from the *Coronaviridae* family^4^. The virus RNA encodes four structural proteins including spike (S), envelope (E), membrane (M), and nucleocapsid (N) proteins, 16 non-structural proteins, and nine accessory proteins^5^. The S glycoprotein consists of an ectodomain (that can be processed into S1 and S2 subunits), a transmembrane domain, and an intracellular domain^6^. Similar to the SARS-CoV, SARS-CoV-2 binds the human angiotensin-converting enzyme 2 (ACE2) via the receptor-binding domain (RBD) within the S1 subunit to facilitate entry into host cells, followed by membrane fusion mediated by the S2 subunit^7–9^

Of the many vaccine platforms, protein subunit vaccines generally have good safety profiles and their production is rapid and easily scalable^10^. Recombinant RBD proteins of SARS-CoV and MERS-CoV have been shown to be immunogenic and induce protective neutralising antibodies in animal models and are therefore considered promising vaccine candidates (reviewed in refs. ^11,12^). RBD from SARS-CoV-2 has recently been confirmed to be inducing neutralising antibodies^13,14^. Recently published studies, including one from our group, found that the majority of the potent neutralising antibodies isolated from SARS-CoV-2-infected patients bound to the RBD^15–17^. We therefore chose to study the immunogenicity of RBD. To improve immunogenicity, we conjugated the RBD onto a VLP. VLP display of protein antigen has been shown to further enhance immunogenicity by facilitating antigen drainage to lymph nodes, enhancing uptake by antigen-presenting cells and increasing B cell receptor crosslinking^10,18^. Moreover, we recently showed that influenza antigens (haemagglutinin (HA) or neuraminidase (NA)) displayed on VLPs (the same VLP used in this study) were highly immunogenic at a low dose (0.1 µg) in mice^18^.

**Fig. 1:**
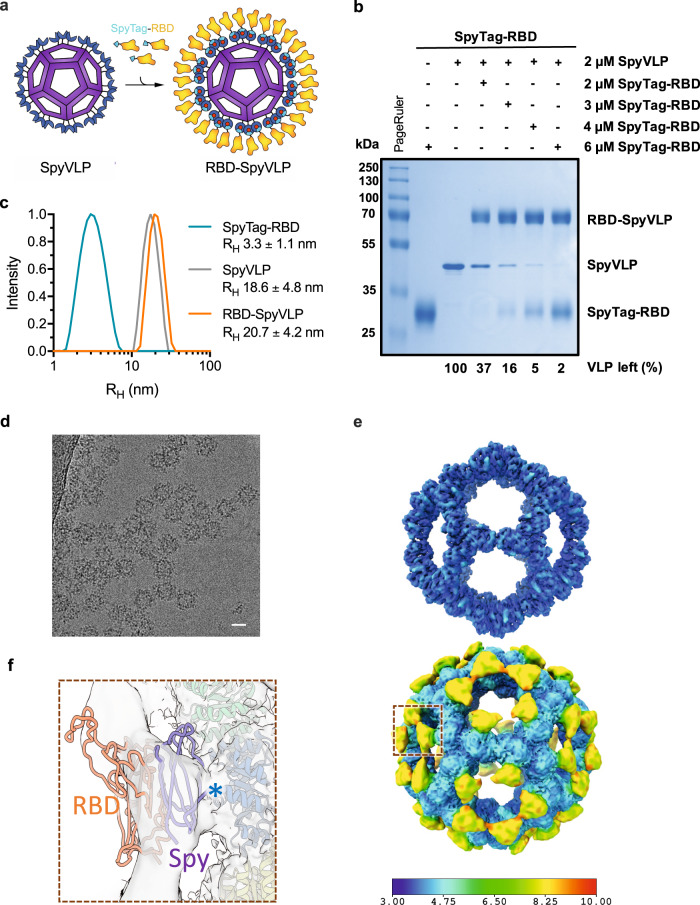
SpyTag-RBD can be efficiently conjugated to SpyCatcher003-mi3 VLP. **a** Schematic diagram of the RBD-SpyVLP vaccine candidate, consisting of SpyCatcher003-VLP conjugated with SpyTag-RBD. The isopeptide bonds formed spontaneously between SpyTag and SpyCatcher003 are indicated with red dots. **b** Conjugation of SpyCatcher003-mi3 with SpyTag-RBD at various ratios. Reactions were performed at 4 °C overnight and analysed using SDS–PAGE with Coomassie staining and densitometry, with the percentage of unreacted VLP shown. Similar reactions have been repeated multiple times during subsequent preparation of material for immunisations. **c** Dynamic light scattering (DLS) characterisation of SpyTag-RBD, SpyVLP, and conjugated RBD-SpyVLP (*n* = 3 individual measurements, values shown as mean ± SD). *R*_H_ hydrodynamic radius. **d** Example of cryo-electron micrograph of RBD-SpyVLP (Scale bar 200 Å). Note that, based on the number of accepted particles following 2D classification, each micrograph contained an average of 25 ‘good’ picked particles. **e** Reconstruction of RBD-VLP with I1 symmetry imposed at two contour levels (top: 0.0428, bottom: 0.2260) coloured by resolution as per the horizontal colour scale (Å). **f** Close up of fit of mi3 model in I1 symmetric map, corresponding to the dark red dashed box in **e** at 0.0428 contour level coloured by monomeric unit at a lower contour such that density presumed to correspond to the SpyTag/SpyCatcher003 and RBD can be seen. The Spy model (PDB 4mli, chain A)^74^ and RBD model (PDB 6yz5)^75^ are labelled, and the N-terminus of mi3 cage (PDB 5kp9^62^ with mutations included) is marked with an asterisk. Source data are provided as a Source Data file.

In the present study, we used the SpyTag/SpyCatcher technology for the assembly of SARS-CoV-2 RBD on a protein nanoparticle platform, SpyCatcher003-mi3^18^. The VLP platform, based on an engineered aldolase from thermophilic bacteria, spontaneously assembles into a hollow 36-nanometre dodecahedral cage with 60 subunits^19,20^. SpyCatcher003 is a variant of SpyCatcher which was engineered for accelerated reaction with SpyTag^21^. The SpyCatcher003-mi3 VLP can be expressed to high yields (100 mg/L of culture media) in *E. coli* and purified using scalable ammonium sulfate precipitation followed by size exclusion chromatography^18^. Furthermore, the VLP platform has high thermal stability and good colloidal properties^18^. Each VLP subunit is genetically fused to SpyCatcher003-protein, allowing efficient decoration of the VLP with SpyTag-fused antigens through covalent isopeptide bonds (Fig. 1a). Previously, SpyTag-mediated VLP decoration has been successfully used for the display of diverse antigens from, e.g. *Plasmodium* spp., influenza A virus, HIV and cancer cells (PD-L1)^18,19,22–24^. Here we show that the RBD-SpyVLP vaccine candidate is highly immunogenic in mice and pigs, inducing robust SARS-CoV-2-neutralising antibody responses. The results of our study demonstrate the potential of the RBD-SpyVLPs as an effective and affordable vaccine for COVID-19 with RBD-SpyVLP being resilient and retaining stability and immunogenicity post-lyophilisation, which will greatly facilitate distribution for vaccination by eliminating cold-chain dependence.

## Results

### RBD can be efficiently displayed on the mi3 VLP via SpyTag/SpyCatcher

To create the RBD-SpyVLP vaccine candidate, the SpyTag (AHIVMVDAYKPTK) coding sequence was fused between the signal sequence from influenza H7 HA (A/HongKong/125/2017) and the N-terminus of the monomeric RBD (amino acid 331–529, NITN…GPKK^6^) (SpyTag-RBD) (see Fig. S1 for the full sequence) and the glycoprotein was expressed in mammalian cells (Expi293F) prior to purification using Spy&Go affinity chromatography^25^. The purified SpyTag-RBD was then conjugated to the SpyCatcher003-mi3 VLP^18,19^ to generate the SpyTag-RBD:SpyCatcher003-mi3 (RBD-SpyVLP) immunogen (Fig. 1a). The SpyTag-RBD can be efficiently conjugated to the SpyCatcher003-mi3 VLP, with 93% display efficiency reached after 16 h (Fig. 1b). This corresponds to an average of 56 RBDs per VLP. We saw no sign of aggregation following coupling and the RBD-SpyVLP is homogeneous, as shown by a uniform peak of the hydrodynamic radius (*R*_H_) at 20.7 ± 4.2 nm in dynamic light scattering (DLS) (Fig. 1c). For immunisation, we chose a conjugation ratio that leaves minimal free RBD (1:1 molar ratio), which corresponds to ~64% display efficiency or around 38 RBD per VLP (Fig. 1b). Cryo-electron microscopic analysis of RBD-SpyVLP cages confirmed the presence of reasonably uniform mi3-cages (Fig. 1d–f). In addition, at low contour levels, the resulting icosahedrally averaged reconstruction, to 3.7 Å resolution (Fig. S2), showed decoration commensurate with the anticipated appended SpyTag-RBD (Fig. 1e, f). The low level of density is expected due to incomplete decoration and likely flexibility of the spacers.

### RBD-SpyVLP is reactive to monoclonal antibodies isolated from recovered patients and is resilient

**Fig. 2:**
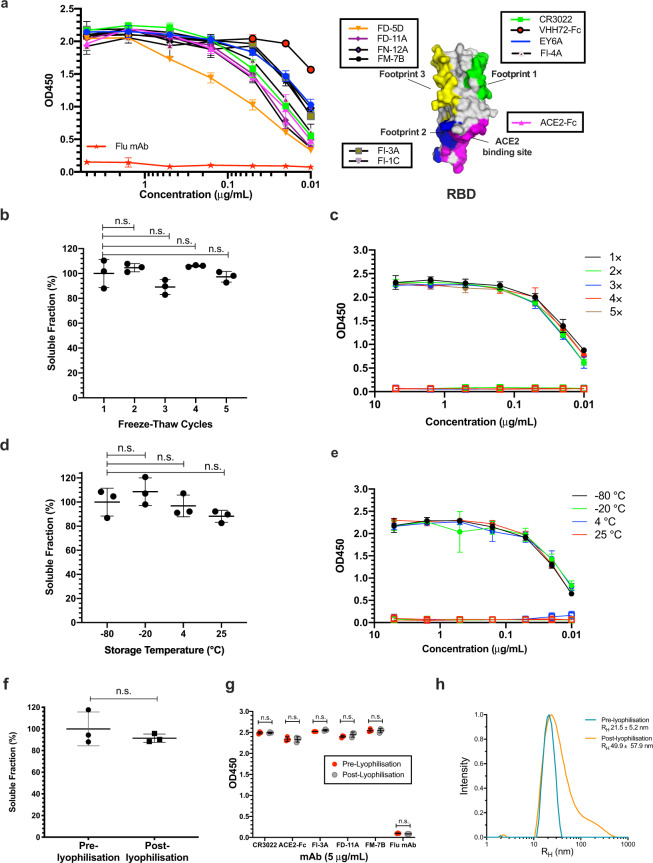
RBD-SpyVLPs are reactive to SARS-CoV-2 specific monoclonal antibodies and ACE2-Fc, and are thermostable and resilient. **a** Binding of RBD-SpyVLP to a panel of monoclonal antibodies isolated from COVID-19 recovered patients that target independent epitopes on the RBD determined using competitive ELISA^26^. Binding of ACE2-Fc, the nanobody VHH72-Fc and an anti-Flu mAb control is also shown. The boxed antibodies form groups that compete with each other and with antibodies or nanobodies with structurally defined footprints: CR3022 (PDB 6W41) (footprint 1), H11-D4-Fc (footprint 2) (PDB 6YZ5), S309 (footprint 3) (PDB 6WPT) and the ACE2-binding site (PDB 6M0J). Each point represents the mean of duplicate readings. The diagram of the RBD, created in PyMOL, shows the ACE2-binding site and the three binding footprints highlighted. **b** Solubility and **c** immunoreactivity of RBD-SpyVLP after freeze−thaw determined using SDS–PAGE and ELISA (CR3022 mAb (closed circles) or an influenza HA mAb (open squares) after one to five cycles of freeze−thawing. **d** Solubility and **e** immunoreactivity of RBD-SpyVLP after storage for 2 weeks at various temperatures, determined using SDS–PAGE and ELISA ((CR3022 mAb (closed circles) or an influenza HA mAb (open squares). **f** RBD-SpyVLP soluble fraction, before and after lyophilisation reconstituted in the same buffer volume and **g** immunoreactivity determined using ELISA with ACE2-Fc and mAbs that target non-overlapping epitopes on the RBD. **h** Dynamic light scattering (DLS) characterisation of RBD-SpyVLP pre- and post-lyophilisation (*n* = 3 independent measurements, values shown as mean ± SD). *R*_H_ hydrodynamic radius. Error bars in **b**, **d** & **f** represent group mean ± SD (*n* = 3 biological independent samples). Error bars in **c**, **e** & **g** represent mean ± 1 SD (*n* = 3 biological independent samples). Statistical difference in **b** & **d** was determined using Kruskal–Wallis test followed by Dunn’s multiple comparison test. Statistical difference in **f** & **g** was determined using Mann–Whitney *U* test. n.s. not significant, two-tailed. Source data are provided as a Source Data file.

To confirm the antigenicity of RBD-SpyVLP, we performed a series of binding assays. Binding to RBD-SpyVLP was tested using a panel of novel monoclonal antibodies (mAbs) some of which are strongly neutralising, from COVID-19-infected donors^26^, that bind to at least three independent epitopes on the RBD. We included the published conformation-specific mAbs (CR3022^27^, S309^28^, EY6A^29^) a nanobody-Fc fusion VHH72-Fc^30^, and a dimeric human ACE2-Fc^31^, for which there are published structures. All tested mAbs and ACE2-Fc bound strongly to the RBD-SpyVLP (Fig. 2a), showing that a broad range of epitopes on the RBD-SpyVLP are exposed and correctly folded. An anti-influenza NA mAb (Flu mAb), used as a negative control, showed no binding to RBD-SpyVLP, confirming the specificity of the assay (Fig. 2a).

**Fig. 3:**
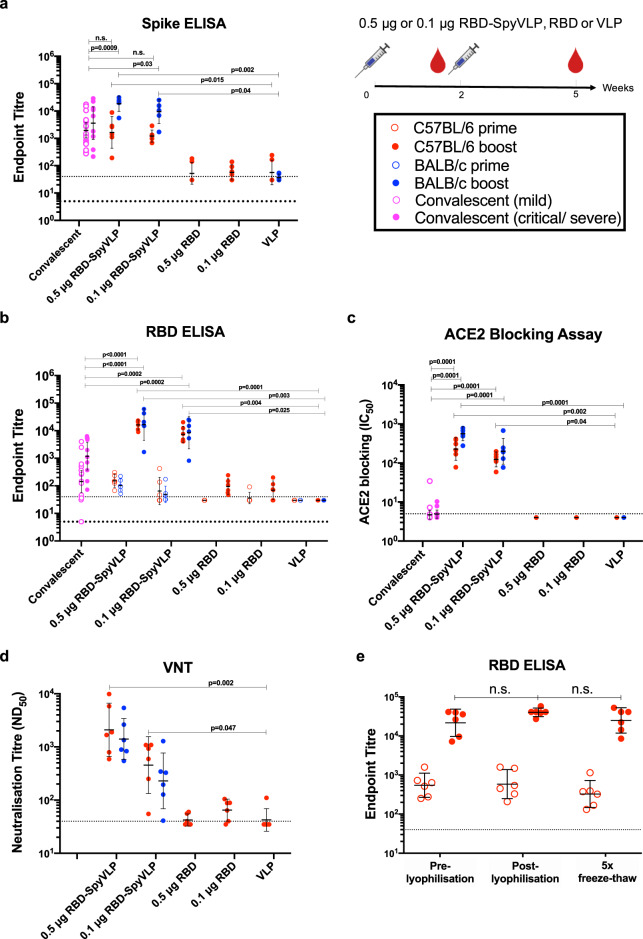
RBD-SpyVLPs induce strong antibody responses in mice that are comparable to the responses in recovered patients. C57BL/6 (red) or BALB/c (blue) mice (*n* = 6 in each group) were dosed twice IM, 2 weeks apart with 0.1 or 0.5 µg purified RBD, RBD-SpyVLP or VLP alone with AddaVax adjuvant added to all. Sera were harvested at 2 weeks after the first dose (open circles) and at 3 weeks after the second dose (closed circles). Sera were analysed in **a** ELISA against full-length spike glycoprotein **b** ELISA against RBD, **c** in an ACE2 competition assay, and **d** in virus neutralisation assays (VNT) against wild-type SARS-CoV-2 virus. **e** Antibody response analysed by RBD ELISA for mice dosed twice with 0.5 µg RBD-SpyVLP (pre-lyophilised, post-lyophilised or freeze–thawed five times). Data are presented as the group geometric means ± 95% confidence intervals. COVID-19 convalescent plasma from humans with mild (open mauve circles) or critical/severe disease (closed mauve circles) were included for comparison. Differences between dosing groups were determined by Kruskal–Wallis test followed by Dunn’s multiple comparison test. Two-tailed Mann–Whitney *U* test was used to compare the immunised groups against convalescent human plasma. Dotted lines represent the lowest mouse sera dilutions tested. Bold dotted line represents the lowest human sera dilution tested. Source data are provided as a Source Data file.

We then tested the stability of RBD-SpyVLP, to determine its resilience and likely sensitivity to failures in the cold-chain^32^. The unconjugated SpyCatcher003-mi3 VLP had previously been shown to be highly thermostable as a platform for antigen display^18^. For conjugated RBD-SpyVLP, we tested its solubility following storage for 2 weeks at −80, −20, 4 or 25 °C in Tris buffered saline (TBS). We then centrifuged out any aggregates and analysed soluble protein by SDS–PAGE with Coomassie staining. We found no significant change in the soluble fraction following storage at 4 °C (*n* = 3, Kruskal–Wallis and Dunn’s post-hoc test, *p* > 0.05), with only a 12% decrease after storage for 2 weeks at 25 °C based on SDS–PAGE/Coomassie staining (Fig. 2d) and no degradation was observed at 25 °C (Fig. S3a). We further analysed the integrity of the sample with ELISA against the conformation-dependent CR3022 mAb and observed no loss of antigenicity under these storage conditions (Fig. 2e). We next assessed the resilience of RBD-SpyVLP to freezing, challenging RBD-SpyVLP with multiple rounds of freeze–thaw. Even after five rounds of freeze–thaw, there was no significant loss of soluble RBD-SpyVLP (Fig. 2b) or CR3022 recognition (Fig. 2c) (*n* = 3, Kruskal–Wallis and Dunn’s post-hoc test, *p* > 0.05) and no degradation was observed based on SDS–PAGE/Coomassie staining (Fig. S3b). After reconstitution following lyophilisation, we saw a minimal change in soluble protein for RBD-SpyVLP (91.5 ± 3.8% of the initial value (mean ± SD) (Fig. 2f), which was not statistically significant (*n* = 3, Mann–Whitney *U* test, *p* > 0.05). There was also no difference in terms of binding of RBD-SpyVLP to a panel of mAbs or ACE2-Fc recognising non-overlapping footprints on the RBD (Fig. 2g). Using DLS, we saw minimal signs of VLP aggregation following lyophilisation (Fig. 2h), which has not affected the solubility or immunogenicity (Fig. 3e) of the RBD-SpyVLP. Overall, RBD-SpyVLP showed a high level of resilience.

### RBD-SpyVLP induces a strong ACE2-blocking and neutralising antibody response in mouse models

We first evaluated the immunogenicity of RBD-SpyVLP in mouse models. C57BL/6 mice (*n* = 6) were immunised intramuscularly (IM) with purified RBD alone (0.1 or 0.5 µg), RBD-SpyVLP (0.1 or 0.5 µg equivalents of the RBD component) or VLP alone, all adjuvanted with AddaVax. AddaVax is a squalene-based oil-in-water nano-emulsion adjuvant, a pre-clinical equivalent to the licensed MF59 adjuvant^33^. Mice were then boosted with the same dose of immunogen 2 weeks later and sera were collected at 3 weeks post-boost. Both the 0.1 and 0.5 µg RBD-only groups showed levels of antibody against RBD or spike glycoprotein only slightly above background, detected using ELISA (serum reciprocal endpoint titre (EPT): 1:94 and 1:68, respectively), and showed no difference compared to the VLP-only group (Fig. 3a, b). Mice immunised with 0.1 and 0.5 µg RBD-SpyVLP groups showed high levels of antibody to RBD (EPT: 0.1 µg: 1:7300, *p* < 0.01 and 0.5 µg: 1:16,117, *p* < 0.001) (Fig. 3a) and to spike (EPT: 0.1 µg: 1:1212 and 0.5 µg: 1:1647, *p* < 0.05) compared to the VLP group (Fig. 3b).

We then tested RBD-SpyVLP in a second mouse strain (BALB/c) with the same dosage regimen to confirm the immunogenicity (Fig. 3a, b). In BALB/c, the 0.1 and 0.5 µg RBD-SpyVLP groups showed higher levels of RBD-specific antibody (EPT: 0.1 µg: 1:8406, *p* < 0.05 and 0.5 µg: 1:16,636, *p* < 0.001) (Fig. 3a) and spike-glycoprotein-specific antibody (EPT: 0.1 µg: 1:9574, *p* < 0.05 and 0.5 µg: 1:18,556, *p* < 0.001) (Fig. 3b) compared to the VLP group.

The ability of immunised mouse sera to block recombinant soluble ACE2 binding to immobilised RBD was then assessed. Sera from the 0.1 and 0.5 µg RBD-SpyVLP immunised C57BL/6 mice had significantly higher ACE2 blocking (IC_50_: 0.1 µg: 1:132, *p* < 0.05 and 0.5 µg: 1:253; *p* < 0.01) activity compared to the VLP-immunised mice (Fig. 3c). C57BL/6 mice immunised with RBD-only showed no detectable ACE2-blocking activity, consistent with the ELISA results (Fig. 3a, b). Similarly, both 0.1 and 0.5 µg RBD-SpyVLP-immunised BALB/c mice had significantly higher ACE2 blocking (IC_50_: 0.1 µg: 1:200 and 0.5 µg: 1:560, *p* < 0.001, respectively) compared to the VLP immunised group (Fig. 3c). In both mouse strains, 0.1 and 0.5 µg RBD-SpyVLP immunised groups had higher antibody titres against RBD (around 5–10-fold; C57BL/6, *p* < 0.0001 and *p* < 0.0001; BALB/c, *p* < 0.01 and *p* < 0.0001) and ACE2 blocking (around 10–50-fold; C57BL/6, *p* < 0.0001 and *p* < 0.0001; BALB/c, *p* < 0.0001 and *p* < 0.0001) compared to plasma donated by patients convalescing from COVID-19 disease (*n* = 28) (Fig. 3b, c). Sera from C57BL/6 mice immunised with 0.1 and 0.5 µg RBD-SpyVLP had comparable spike glycoprotein-specific antibody responses compared to convalescent humans whereas sera from BALB/c mice immunised with either dose had higher spike glycoprotein-specific antibody responses compared to convalescent humans (around 2-fold, 0.1 µg, *p* < 0.05 and 0.5 µg, *p* < 0.001) (Fig. 3a, b).

The antibody response in mice was assessed for neutralisation potency using a live SARS-CoV-2 virus (hCoV-19/England/02/2020, EPI_ISL407073) neutralisation assay (VNT) based on virus plaque reduction. Sera from C57BL/6 mice immunised with either dose of unconjugated RBD showed low level neutralising titres (ND_50_ 1:59 and 1:32, *p* > 0.05) compared to the RBD-SpyVLP group, again consistent with ELISA and ACE2-blocking activity (Fig. 3d). Both 0.1 and 0.5 µg RBD-SpyVLP-immunised groups exhibited high neutralising titres, for both C57BL/6 (ND_50_: 1:450–1:2095) and BALB/c mice (ND_50_: 1:230–1:1405) (Fig. 3d). Consistent neutralising activity in sera from C57BL/6 mice was found using a VNT in an independent laboratory with the hCoV-19/VIC01/2020 isolate (GenBank MT007544) (Table S1).

We tested the immunogenicity of RBD-SpyVLP post-lyophilisation or after five freeze–thaw cycles. C57BL/6 mice (*n* = 6) were immunised IM with 0.5 µg RBD-SpyVLP (pre-lyophilised, post-lyophilised or 5× freeze–thaw) and sera were harvested post prime and post boost and tested for binding in a RBD ELISA. There was no difference between the immune responses in all three groups tested (Fig. 3e) (Kruskal–Wallis, followed by Dunn’s post-hoc test, *p* > 0.05), showing that lyophilisation and freeze–thaw had not compromised the immunogenicity of the RBD-SpyVLP.

### RBD-SpyVLP is highly immunogenic and induces strong neutralising antibody response in pigs

We tested RBD-SpyVLP for its immunogenicity in pigs as a large, genetically outbred animal model. Pigs have previously been reported to be a reliable model to study vaccines for use in humans because of their highly similar physiologies and immune systems^34,35^. The pig model has been used recently to test an adenovirus vector vaccine candidate against SARS-CoV-2 (ChAdOx1 nCoV19)^36^. We immunised pigs (*n* = 3) with a dose of RBD-SpyVLP that we intend to use in humans (5 µg) or with a 10-fold higher dose (50 µg) to study dose-response. A third group (*n* = 3) receiving 100 µg of purified trimeric spike glycoprotein (spike) was included as a control. Pigs were immunised IM twice, 28 days apart, with 5 or 50 µg RBD-SpyVLP or 100 µg of spike, all adjuvanted with AddaVax.

**Fig. 4:**
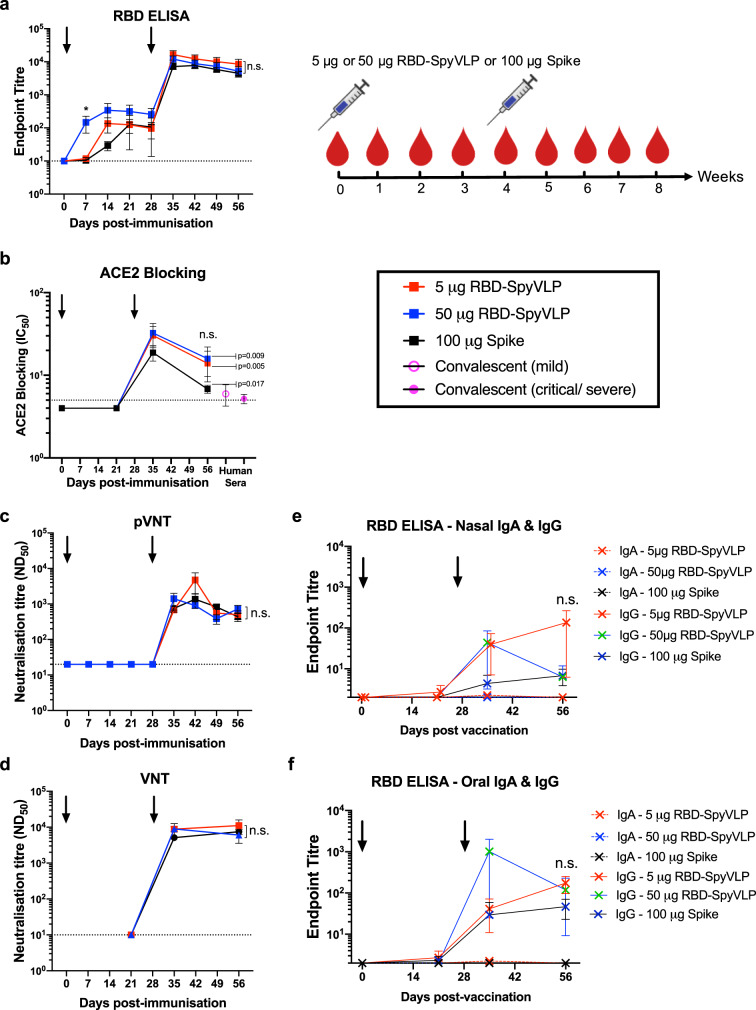
RBD-SpyVLPs induce persistent and strong neutralising antibody responses in pigs. Pigs (*n* = 3 in each group) were dosed twice (prime and boost) IM, 4 weeks apart with 5 or 50 µg RBD-SpyVLP or 100 µg of spike glycoprotein, all adjuvanted with AddaVax and sera were harvested at indicated time-points and analysed in **a** ELISA against RBD, **b** in an ACE2 competition assay, **c** in pseudovirus neutralisation assay (pVNT), and **d** wild-type virus neutralisation assay (VNT). Antibody levels in **e** nasal and **f** oral swabs were measured at indicated time points using RBD ELISA. Data are presented as the group mean ± 1 SEM. Black arrows indicate when the vaccines were administered. Kruskal–Wallis test was used to compare data on day 56 in **b**–**e**, n.s. not significant. *p < 0.05 and data on day 56 were compared to convalescent human plasma using two-tailed Mann–Whitney *U* Test. Dotted lines represent the lowest dilutions of sera tested in the assays. Source data are provided as a Source Data file.

As early as day 7, pigs immunised with 50 µg RBD-SpyVLP showed a detectable anti-RBD antibody response, whereas no antibody response was detected at day 7 for 5 µg RBD-SpyVLP or 100 µg spike groups (Fig. 4a) (*p* < 0.05, Kruskal–Wallis test). The antibody response in all three groups increased gradually to day 14 when the response reached a plateau. The group receiving 50 µg RBD-SpyVLP showed a trend of slightly higher antibody response than the other two groups until the day of boost (day 28) (Fig. 4a). The antibody response in all three groups increased by around 100-fold one week after boosting and remained high until at least day 56. There was no dose-response difference between the 5 and 50 µg RBD-SpyVLP groups (*p* > 0.5, Kruskal–Wallis test) for day 56 data (Fig. 4a).

ACE2-blocking activity in the pig serum was measured at day 21, day 35 and day 56. No ACE2 blocking was detected at day 21, prior to the booster dose. ACE2 blocking was detected post-boost at day 35 (IC_50_ 1:10–1:50) and reduced at day 56 (IC_50_ 1:5–1:25) in all three groups (Fig. 4b). ACE2-blocking activities in all three groups were significantly higher than for sera from convalescent humans (5 µg RBD-SpyVLP, *p* < 0.01; 50 µg RBD-SpyVLP, *p* < 0.01; 100 µg, *p* < 0.05), by around ~3–6-fold (Kruskal–Wallis test followed by Dunn’s posthoc test on day 56 data).

Neutralising antibody responses in the pigs were assessed against both SARS-CoV-2 pseudotyped lentivirus and live SARS-CoV-2 virus. All three groups exhibited similar neutralisation titres against pseudovirus (ND_50_: ~1:500–1:4000) after boost which remained until at least day 56, with no difference between the three groups (*p* > 0.05, Kruskal–Wallis test on day 56) (Fig. 4c). Similarly, no difference in neutralising activity was detected in all three groups against live virus at day 21 (Fig. 4d). Neutralisation was detected on both day 35 and 56 in all groups, with ND_50_ levels between 1:6000 and 1:11,000 on day 56 and no significant difference between the three groups (*p* > 0.05, Kruskal–Wallis test on day 56 data) (Fig. 4d). Nasal and oral secretions were also tested for the presence of neutralising antibodies: here RBD-specific IgG, but notably not IgA, was detected in all three groups with no significant difference between groups post-boost for both nasal and oral secretions (*p* > 0.05, Kruskal–Wallis followed by Dunn’s Post-hoc) (Fig. 4e, f).

We assessed the levels of RBD-specific B cells in peripheral blood over the course of vaccination. The predominance of an IgG response following the booster immunisation was confirmed in all three groups by assessment of RBD tetramer labelling of IgM+, IgG+ and IgA+ B cells in peripheral blood and IgG ELISpot assay (Fig. S4a). Significant increase in the frequency of RBD-tetramer binding IgG+ cells were observed on day 31, 33 and 35 post-immunisation (3, 5 and 7 days post-boost) (*p* < 0.05), which were not significantly different between the spike and the RBD-SpyVLP groups. Significant increases in the frequency of B cells secreting RBD-specific IgG were detected by ELISpot assay in both RBD-SpyVLP groups on day 31 and 33 (*p* < 0.05) (Fig. S4b). A far smaller increase in IgM+ cells binding to RBD-tetramer was observed in the 50 µg RBD-SpyVLP group on day 33 which was significantly greater than the 5 µg RBD-SpyVLP and spike groups (*p* < 0.05) (Fig. S4a). No significant changes were seen in the frequency of IgA+ B cells binding to RBD at any timepoints compared to pre-immunisation. We also performed longitudinal analysis of CD4+ and CD8+ T cell responses following immunisation (Fig. S5a). Intracellular cytokine staining of S-peptide-stimulated peripheral blood mononuclear cells (PBMCs) demonstrated a weak T cell IFN-γ response. Only the CD4+ T cell response of the 5 µg RBD-SpyVLP groups on day 35 was statistically significant compared to pre-immunisation (*p* < 0.05) (Fig. S5a). CD8+ T cell responses were not statistically significant at any timepoints compared to pre-immunisation.

### RBD-SpyVLP induces polyclonal antibody responses against the RBD in mice and pigs

**Fig. 5:**
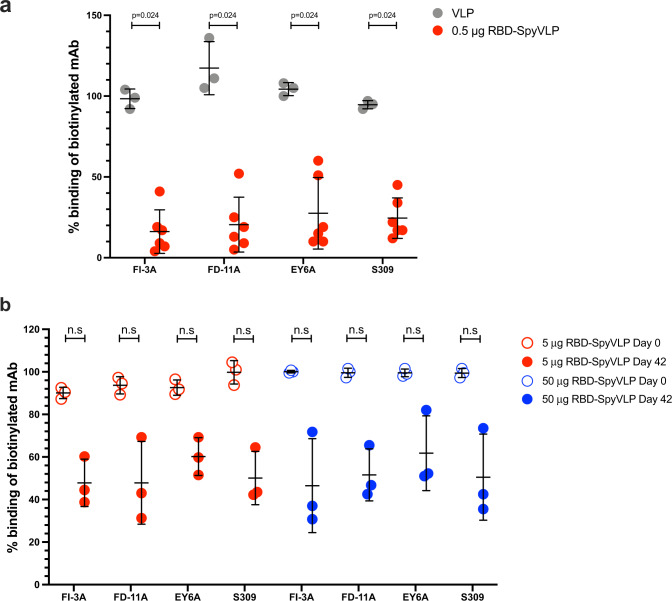
RBD-SpyVLP immunisation of mice and pigs elicits polyclonal antibody responses that target all key epitopes on the RBD. Competition ELISA of four human mAbs that target three different key epitopes on the RBD with post-boost BALB/c sera (0.5 µg RBD-SpyVLP or VLP alone control) (**a**). Competition ELISA of four human mAbs that target three different key footprints (EY-6A footprint 1, FD-11A footprint 2, FD-11A footprint 3, S309 footprint 3) on the RBD with post-boost pig sera (5 or 50 µg RBD-SpyVLP) compared to preimmune sera (day 0) (**b**). Each point in **a** represents an average of duplicate readings of a serum sample from one animal 3 weeks post boost tested at 1:20 dilution. Each point in **b** represents an average of quadruplicate readings of a serum sample from one animal on day 42 tested at 1:20 dilution. Data are presented as group means ± 1 SD. Statistical differences were determined by two-tailed Mann–Whitney *U* test. n.s. not significant. Source data are provided as a Source Data file.

A concern regarding RBD-based vaccines is whether the immune response will be focussed to a single site on the antigen, because of the relatively small size of the RBD, potentially leading to a narrow response that would be sensitive to immune escape^37,38^. A recent report showed that passage of virus exposed to a single neutralising mAb led to selection of escape mutant of SARS-CoV-2 in vitro, whereas a cocktail of two neutralising antibodies to independent epitopes prevented emergence of neutralisation-escape mutations, demonstrating the importance of a polyclonal antibody response^39^. We assessed sera from mice and pigs immunised with RBD-SpyVLP for antibody responses that target multiple RBD epitopes using a competition ELISA against four different mAbs: FI-3A, FD-11A, EY6A and S309, that target three non-overlapping epitopes on the RBD with FD-11A and S309 showing overlap as defined by competition ELISA (see diagram in Fig. 2a)^26,28,29^. BALB/c mice immunised with RBD-SpyVLP showed competition against all four mAbs tested (Fig. 5A). BALB/c mice immunised with VLP-only showed no competition (*p* < 0.05, Mann–Whitney *U*-test) (Fig. 5a). A similar pattern was observed in the C57BL/6 mice (Fig. S6). Comparison of preimmune and day 42 post-RBD-SpyVLP-immunisation sera from pigs showed a trend of partial competition against all four mAbs, but this was not statistically significant because of inter-animal variation (Fig. 5b). When responses of individual pigs were compared to their pre-immune sera, 2 out of 3 animals in each dosing group showed significant competition for the antibodies FI-3A and S309 that defined independent neutralising epitopes compared to their preimmune sera (Fig. S7). These results show that RBD-SpyVLP does not have an immunodominant epitope and does not induce a highly focussed antibody response, making it a vaccine candidate that is likely to resist the generation of neutralisation-escape mutants.

## Discussion

In the current study, we investigated an RBD-based VLP vaccine candidate for COVID-19 based on SpyTag/SpyCatcher technology which was used to assemble RBDs into the mi3 VLP via the formation of an irreversible isopeptide bond^22^. We showed RBD-SpyVLPs to be strongly immunogenic in mice and pigs, inducing high titre neutralising antibody responses against wild type SARS-CoV-2 virus. This study confirms that the RBD is the key immunogenic domain for eliciting neutralising polyclonal antibodies against SARS-CoV-2, in line with studies showing that highly neutralising antibodies isolated from convalescent patients bind to the RBD^15–17,26,28,40–45^. We showed that RBD-SpyVLPs are recognised by a panel of mAbs isolated from convalescent patients^26^ binding to various epitopes on the RBD (Fig. 2a). This distributed reactivity shows that the key epitopes that could potentially induce protective antibodies to RBD are present in RBD-SpyVLPs.

We detected negligible antibody responses in mice vaccinated with equivalent doses (0.1 or 0.5 µg) of purified RBD alone, but strong responses to the RBD when displayed on the VLP (Fig. 3). Previous studies showed that RBD from SARS-CoV and SARS-COV-2 can induce neutralising antibodies in animal models but typically after administration of much higher doses (e.g. ~50 to 100 µg) and with frequent dosing^13,14,46^. On the other hand, we showed that high titre neutralising antibody responses can be detected in two strains of mice immunised with relatively low doses of RBD-SpyVLP (to ND_50_ ~500 to 2000). These results confirm the enhanced immunogenicity of RBD when displayed on SpyVLPs. Sera from mice immunised with both 0.1 or 0.5 µg of RBD-SpyVLP exhibited high levels of antibody against SARS-CoV-2 RBD and full-length spike glycoprotein and ACE2 blocking activity (Fig. 3b–d). All of these responses were higher than the levels found in plasma from convalescent humans. Together, these observations suggest that RBD-SpyVLP vaccination could potentially elicit protective antibody responses against SARS-CoV-2 in humans.

RBD-SpyVLP vaccination also induces high titre neutralising antibody responses in pigs (ND_50_ ~1:11,000) with a dose that we aim to use for subsequent human trials (5 µg) (Fig. 4d). At a dose around 2-fold less (based on molar ratio), 5 µg of RBD-SpyVLP induced similar neutralisation titres compared to 100 µg of spike glycoprotein, showing the potent immunogenicity of RBD-SpyVLP vaccination. Transudate RBD-specific IgG from serum can be detected in the oral and nasal cavities (Fig. 4e, f). Surprisingly, no increase in antibody titre was observed in pigs that received a higher dose of antigen (50 µg RBD-SpyVLP). There was a trend that the 50 µg RBD-SpyVLP group generated a more rapid and higher response post-prime but the antibody response between the 5 and 50 µg RBD-SpyVLP groups were identical post-boost. This suggests a threshold effect.

Since RBD-SpyVLPs induce antibody responses that target multiple epitopes on the RBD the chance of selecting neutralisation-escape mutants should be greatly reduced. Circulating SARS-CoV-2 stains are constantly mutating and the likelihood of persistence of the virus in the human population is high^47^.

Antibody-dependent inhibition of binding of the RBD of the spike protein to its ACE2 receptor has been correlated with the neutralising titre in convalescent human sera^15–17,40,41,45,48^. We observed differences in the levels of ACE2 blocking in sera from immunised mice and pigs (Figs. 3c and 4b). Sera taken from mice immunised with RBD-SpyVLP had at least one order of magnitude higher ACE2-blocking activity compared to serum from pigs, despite neutralisation titres being comparable (Figs. 3d and 4d). This suggests that mice and pigs may produce distinct antibody responses against the vaccine candidate, although they were equally potent in neutralising live viruses. Surprisingly, ACE2 blocking in both mild and critical/severe convalescent humans who had natural infection were also low compared to the sera from immunised mice (Fig. 3c). Similarly low levels of ACE2-blocking antibodies have been detected in a proportion of sera from humans^16^.

Nevertheless, the RBD-SpyVLP induces strong neutralising antibody responses in both mice and pigs. The potential for a vaccine based on the RBD is further emphasised by the sterile immunity induced in non-human primates (NHP) (*Macaca mulatta*) by two doses of 20–40 µg of unconjugated RBD in Al(OH)_3_ adjuvant^14^, and the successful induction of high titre neutralising antibody responses with an elegant self-assembling RBD-virus like nanoparticle^49^. Recently published papers demonstrated the superior immune response elicited by spike-based VLPs compared to spike protein alone, and that immunity to up to eight different RBDs can be induced simultaneously by SpyCatcher003-mi3 VLPs coated with a mosaic of RBDs of varied viral origin^50,51^.

A recently published report on an inactivated SARS-CoV-2 vaccine candidate showed that vaccinated NHP with a serum neutralising titre (ND_50_) of <1:100 were still protected against wild type SARS-CoV-2 challenge with no weight loss and no detectable lung pathology^52^. In our study, RBD-SpyVLP-vaccinated mice and pigs had ND_50_ at least an order of magnitude higher than 1:100, which would be expected to provide protection. Sera from pigs immunised with RBD-SpyVLP had similar, if not higher, neutralisation titres against wild type SARS-CoV-2 compared to pigs immunised by adenoviral vector (ChAdOx1 nCoV-19; both vaccines given as two doses in the same timeframe)^36^. A single dose of ChAdOx1 nCoV-19 has been shown to be protective against viral pneumonia and lung pathology following SARS-CoV-2 challenge (ND_50_ ~1:20) in NHP, but did not prevent virus shedding in the nasal cavity^53^. The seemingly similar ND_50_ of the RBD-SpyVLP compared to the ChAdOx1 nCoV-19 in pigs after two doses suggests that the RBD-SpyVLP will be as protective as the ChAdOx1 nCoV-19 in NHP and most likely in humans if similar neutralisation titres are achieved. A pre-stabilised spike glycoprotein vaccine candidate, NXV-CoV2273 developed by Novavax, showed neutralising antibody responses in humans that were 4-fold higher than convalescent human sera, when given in two doses of 5 µg with Matrix M1 adjuvant^54^. In light of the higher efficacy of RBD-SpyVLP compared to spike glycoprotein in pigs (Fig. 4), we are hopeful that at least similar efficacy of RBD-SpyVLP could be achieved in humans.

Recently published studies reveal the relatively short-lived antibody response to SARS-CoV-2 in convalescent patients^55,56^. Further work is required to define the significance of these results for the longevity of protective immunity. In a human cohort, decline in the absolute level of antibody in the circulation was shown to be balanced by improved efficiency of neutralisation through probable affinity maturation^16^. It is also possible that re-infection with the virus would lead to a rapid strong protective secondary antibody response from memory B cells, despite reduced levels of circulating antibody. Here, we have shown that the antibody response in pigs immunised with RBD-SpyVLP persisted for at least 2 months and remained neutralising. Studies on the longevity of the immune responses are to be undertaken in the near future.

Apart from being highly immunogenic, SpyVLPs provide a versatile modular vaccine platform to facilitate conjugation with other antigens. Should a mutation in the RBD arise in circulating SARS-CoV-2 strains^47^, a matched RBD-SpyVLP could be manufactured rapidly. In addition, more than one RBD variant can be co-displayed on the VLP, to provide broader protection against various SARS-CoV-2 strains^50,57^. The RBD-SpyVLP can also be co-displayed with antigens from other pathogens such as the HA and NA from influenza virus. We have recently shown HA and NA to be highly immunogenic in mice after formulation as a SpyVLP^18^. This assembly could potentially provide protection against both SARS-Cov-2 and influenza viruses. Testing resilience of the vaccine candidate, we found that RBD-SpyVLP is stable at ambient temperature, resistant to freeze–thaw, and can be lyophilised and reconstituted with minimal loss in activity (Fig. 2b–g) or immunogenicity (Fig. 3e). This resilience may not only simplify vaccine distribution worldwide, especially to countries where cold-chain resources are incomplete, but also reduce the overall vaccine cost by removing cold-chain dependence. We are currently investigating cheaper and more scalable alternatives to produce RBD-SpyVLP to cope with the global demand for a SARS-CoV-2 vaccine. Collectively, our results show that the RBD-SpyVLP is a potent and adaptable vaccine candidate for SARS-CoV-2.

## Methods

### Expression constructs

The SpyTag-RBD expression construct (Fig. S1) consists of influenza H7 HA (A/HongKong/125/2017) signal-peptide sequence, SpyTag^58^, (GSG)_3_ spacer and SARS-CoV-2 spike glycoprotein (GenBank: NC045512) (amino acid 331–529, NITN…GPKK^6^). The insert was ordered from GeneArt and subcloned into pcDNA3.1 expression plasmid using unique *Not*I-*EcoR*I sites to create pcDNA3.1-SpyTag-RBD (Addgene 159999, GenBank MT945427) (see Fig. S1). pET28a-SpyCatcher003-mi3 (Addgene 159995, GenBank MT945417) was created by replacing SpyCatcher in pET28a-SpyCatcher-mi3 with SpyCatcher003^18^. RBD used in the RBD ELISA was expressed from a codon optimised RBD cDNA subcloned into the vector pOPINTTGNeo incorporating a C-terminal His_6_ tag (RBD-6H) as previously described^36^. Human ACE2 fused to human IgG1 Fc domain (ACE2-Fc) used in the ACE2 competition ELISA was expressed from codon optimised human ACE2 cDNA (amino acids 19–615) fused to the Fc region and a C-terminal His_6_ tag subcloned into the vector pOPINTTGNeo.

### Expression and purification of SpyCatcher003-mi3

SpyCatcher003-mi3 was expressed in *E. coli* BL21(DE3) RIPL cells (Agilent) as previously described^18^. Heat-shock transformed cells were then plated on LB-Agar plates (50 µg/mL kanamycin) and incubated for 16 h at 37 °C. A single colony was picked and cultured in 10 mL starter LB culture (50 µg/mL kanamycin) for 16 h at 37 °C and shaking at 200 rpm. The preculture was diluted 1:100 into 1 L LB (50 µg/mL kanamycin and 0.8% (w/v) glucose) and cultured at 37 °C, 200 rpm until OD600 ~0.6. Protein expression was induced with isopropyl β-d-1-thiogalactopyranoside (IPTG) (420 µM) and incubated at 22 °C, 200 rpm for a further 16 h. The culture was centrifuged and the pellet was resuspended in 20 mL 25 mM Tris–HCl, 300 mM NaCl, pH 8.5 with 0.1 mg/mL lysozyme, 1 mg/mL cOmplete mini EDTA-free protease inhibitor (Merck) and 1 mM phenylmethanesulfonyl fluoride (PMSF). Cell suspension was incubated at 22 °C for 30 min on a platform shaker and sonicated on ice four times for 60 s at 50% duty-cycle using an Ultrasonic Processor (Cole-Parmer). Cell lysate was clarified at 35,000 × *g* for 45 min at 4 °C. The supernatant was filtered through 0.45 and 0.22 µm syringe filters (Starlab) and 170 mg ammonium sulfate was added per mL of lysate. SpyCatcher003-mi3 particles were precipitated by incubating the lysate at 4 °C for 1 h while mixing at 100 rpm. Precipitated particles were pelleted by centrifugation at 30,000 × *g* for 30 min at 4 °C. The collected pellet was resuspended into 8 mL TBS pH 8.5 (25 mM Tris–HCl, 150 mM NaCl). Residual ammonium sulfate was removed by dialysing for 16 h against 500-fold excess of TBS. Dialysed SpyCatcher003-mi3 was concentrated to 4 mg/mL using a Vivaspin 20 100 kDa spin concentrator (Vivaproducts) and centrifuged at 17,000 × *g* for 30 min at 4 °C to pellet any insoluble material. The supernatant was filtered through a 0.22 µm syringe filter. The purified SpyCatcher003-mi3 was then further purified using size-exclusion chromatography (SEC). In brief, 2.5 mL was loaded into a HiPrep Sephacryl S-400 HR 16-600 SEC column (GE Healthcare) equilibrated with TBS using an ÄKTA Pure 25 system (GE Healthcare). Proteins were separated at 1 mL/min while collecting 1 mL elution factions. The fractions containing the purified particles were identified by SDS–PAGE, pooled, and concentrated using a Vivaspin 20 100 kDa MW cut-off centrifugal concentrator. Endotoxin was removed from the SpyCatcher003-mi3 samples using Triton X-114 phase separation as previously described^18^. The concentration of endotoxin-depleted particles was measured using bicinchoninic acid (BCA) assay (Pierce) and particles were stored at −80 °C.

### Expression and purification of SpyTag-RBD

SpyTag-RBD was expressed in Expi293F cells using ExpiFectamine293 transfection reagent (Thermo Fisher) according to the manufacturer’s protocol. Supernatant was harvested between 5 and 7 days post transfection and filtered through a 0.22 µm filter, before purifying using Spy&Go affinity purification with minor modifications^18,25^. Briefly, filtered supernatants were diluted with one-third supernatant volume of TP buffer (25 mM orthophosphoric acid adjusted to pH 7.0 at 22 °C with Tris base) and adjusted to pH 7. Spy&Go resin in same buffer was mixed with the diluted supernatant and incubated at 4 °C for 1 h with gentle agitation. The mixture was poured into an Econo-Pak column (Bio-Rad) and allowed to empty by gravity. The resin was washed with 2 × 10 column volumes of TP buffer and SpyTag-RBD was eluted with 2.5 M imidazole in TP buffer adjusted to pH 7.0 at room temperature (RT). One column volume of elution buffer was added to the resin at a time and incubated for 5 min before collecting each fraction. Elution fractions were analysed using SDS–PAGE with Coomassie staining and the fractions containing SpyTag-RBD were pooled and dialysed against 10 mM Tris–HCl pH 8.0 with 200 mM NaCl. The sample was concentrated using Vivaspin-20 10 kDa and further purified via SEC using ÄKTA Pure 25 (GE Life Sciences) equipped with Superdex 75 pg 16–600 column (GE Life Sciences), run at 1 mL/min. The dialysis buffer was used as the mobile phase. The final yield of purified SpyTag-RBD was around 100 mg/L. The heterogeneity in the SpyTag-RBD band on SDS–PAGE is expected from the presence of different glycoforms on the N-linked glycosylation sites in the construct (Fig. S1).

### RBD-SpyVLP conjugation

SpyTag-RBD at 2, 3, 4 or 6 µM was conjugated at 4 °C for 16 h with 2 µM SpyCatcher003-mi3 (VLP:RBD ratio 1:1, 1:1.5, 1:2 and 1:3) in TBS pH 8.0. Possible aggregates were then removed by centrifugation at 16,900 × *g* for 30 min at 4 °C. Samples of the supernatant were mixed with reducing 6× loading dye (0.23 M Tris–HCl, pH 6.8, 24% (v/v) glycerol, 120 μM bromophenol blue, 0.23 M SDS, 0.2 M dithiothreitol) and resolved on 12%, 14% or 16% SDS–PAGE using the XCell SureLock system (Thermo Fisher). Gels were then stained with InstantBlue Coomassie (Expedion) and imaged using ChemiDoc XRS imager (Bio-Rad). The intensities of bands on each lane were quantified using ImageLab (version 5.2) software (Bio-Rad) and Fiji distribution of ImageJ (version 1.51n). Conjugation efficiency (as % of unconjugated SpyVLP left) was calculated as 100*(band density of unconjugated SpyVLP left in the conjugation reaction/band density of SpyVLP only control (2 μM)).

### RBD-SpyVLP thermostability and lyophilisation tests

30 μL of RBD-SpyVLP in TBS stored in thin-walled PCR tubes were subjected to freeze–thaw cycles (one to five cycles) by storing the tubes in a −80 °C freezer for 15 min or until the whole tube had frozen over, followed by incubation at RT for 10 min. For the storage temperature study, 30 μL of RBD-SpyVLP in TBS stored in thin-walled PCR tubes were incubated at −80, −20, 4 °C or RT (25 °C) for 14 days. The RBD-SpyVLP samples were then resolved on 4–12% Tris–Bis SDS–PAGE (Thermo Fisher) and analysed by densitometry following Quick Coomassie (Generon) staining. All samples were analysed in triplicate and plotted as mean ± 1 standard deviation (SD). The sample stored at −80 °C for 2 weeks, which had been through only 1 freeze–thaw cycle, was defined as 100% soluble. For lyophilisation, 100 μL of RBD-SpyVLP (125.5 μg/mL) in TBS pH 8.0 prepared in a Protein LoBind microcentrifuge tube (Fisher Scientific) was stored in a -80 °C freezer and then cooled with liquid nitrogen before lyophilisation. A BenchTop 2K freeze-dryer (VirTis) was used for 24 h at 0.14 mbar and −72.5 °C to freeze–dry the sample. Lyophilised sample was reconstituted in the same original volume (100 μL) of MilliQ water and centrifuged at 16,900 × *g* for 30 min to remove aggregates, before analysis with SDS–PAGE or ELISA. For testing of RBD-SpyVLP on ELISA, 50 μL of RBD-SpyVLP samples diluted in PBS (137 mM NaCl, 2.7 mM KCl, 10 mM Na_2_HPO_4_, 1.7 mM KH_2_PO_4_, pH 7.4) to 0.5 μg/mL were coated on NUNC plates at 4 °C overnight, washed with PBS, and blocked with 300 μL of 5% (w/v) skimmed milk in PBS for 1 h at RT. Plates were then washed and incubated with 50 μL CR3022 (10 μg/mL) antibody (for freeze–thaw and storage temperature study) or a panel of mAbs as indicated in the graph (5 μg/mL) (for the lyophilisation study) diluted in PBS/0.1% (w/v) BSA for 1 h at RT. Plates were washed and incubated with horse radish peroxidase (HRP)-conjugated goat-anti-human IgG antibody (Dako, P0214) (diluted 1:1600 in PBS/0.1% (w/v) BSA) for 1 h at RT. Plates were then washed and developed with 50 μL of POD 3,3′,5,5′-tetramethylbenzidine (TMB) substrate (Roche) for 5 min and stopped with 50 μL of 1 M H_2_SO_4_. Absorbance was measured on a Clariostar plate reader (BMG Labtech). To test the reactivity of RBD-SpyVLP against a panel of anti-SARS-CoV-2 RBD antibodies, 50 μL of RBD-SpyVLP samples diluted in PBS to 0.5 μg/mL were coated on NUNC plates at 4 °C overnight. Plates were then washed and blocked with 300 μL of 5% (w/v) skimmed milk in PBS for 1 h at RT. Plates were washed and incubated with antibodies diluted in PBS with 0.1% (w/v) BSA in a 2-fold dilution series in 50 μL for 1 h. Second layer antibody was added as described above and plates were developed as above.

### Dynamic light scattering

Samples were centrifuged for 30 min at 16,900 × *g* at 4 °C to pellet possible aggregates. Before each measurement, the quartz cuvette was incubated in the instrument for 5 min to stabilise the sample temperature. Samples were measured at 125–250 µg/mL total protein concentration. 30 µL of sample was measured at 20 °C using an Omnisizer (Victotek) with 20 scans of 10 s each. The settings were 50% laser intensity, 15% maximum baseline drift, and 20% spike tolerance. The intensity of the size distribution was normalised to the peak value and plotted in GraphPad Prism 8 (GraphPad Software).

### Cryo-EM

A 3 μL aliquot of RBD-SpyVLP sample at ~0.9 mg/mL (determined by Nanodrop) was applied to a freshly glow-discharged (high intensity for 30 s Plasma Cleaner PDC-002-CE, Harrick Plasma) copper quantifoil 2/1-200 mesh grid. Cryo grid preparation used a Vitrobot Mark IV (Thermo Fisher). Excess liquid was removed by blotting for 6 s with a force of +6 using vitrobot filter paper (grade 595, Ted Pella Inc.) at 5.5 °C, 100% relative humidity, before plunging into liquid ethane. Data were collected using Titan Krios G2 (Thermo Fisher) operating at 300 kV with a K2 camera and a GIF Quantum energy filter (Gatan) with a 30 eV slit at a nominal magnification of 165,000-fold, corresponding to a calibrated pixel size of 0.82 Å per pixel. To prepare the example micrograph shown in Fig. 1d, a movie with a total dose of 48.1e^−^/Å^2^ applied across 40 frames was collected using SerialEM software^59^ (see Table S2 for data collection parameters). Motion and contrast transfer function (CTF) correction of raw movies were performed on the fly using cryoSPARC live patch-motion and patch-CTF correction. Poor-quality images were discarded after manual inspection of CTF and motion estimations. Particles were then blob-picked in cryoSPARC v.2.14.1^60^ and after inspection of 2D classes, classes of interest were selected to generate templates for complete particle picking. A total number of 3,163,892 particles were picked using a template derived from 65,727 blob-picked particles and a final number of 156,785 particles were selected after 2D classification followed by 3D classification using three ab-initio models with no symmetry applied.

The best class was then refined with I1 symmetry using a tight mask around the cage protein unit to avoid map distortion due to SpyTag-RBD signal dominating alignment. In such cases where a custom mask (i.e. not automatically generated by cryoSPARC) was designed, initial masks were created by first using the ‘colorzone’ option with a 8 Å radius in Chimera^61^ and appropriate PDB models to extract the desired volume. The resulting volume was then imported into relion and a mask created without extension before being imported as a mask in cryoSPARC. Non-uniform refinement with icosahedral (I1) symmetry imposed followed by local filtering resulted in a map with estimated resolution of 3.86 Å, gold standard Fourier shell correlation (FSC) = 0.143 (Fig. S2). In parallel, the same particle set was locally refined (default C1 symmetry in CryoSPARC), yielding a map with an estimated resolution of 4.98 Å (gold standard FSC = 0.143). For both I1 and C1 reconstructions, density could be accounted for by the mi3 nanocage scaffold. At lower contour levels, again with and without symmetry, additional density surrounding the five-fold axis that could accommodate the RBD and SpyTag/SpyCatcher003 was observed (Fig. 1e, f). Structures were modelled by rigid body fitting of PDB ID, 5kp9^62^ adjusted to contain the Cys to Ala mutations present in the mi3 cage scaffold using UCSF Chimera^61^ and Coot^63^. Figures were prepared using ChimeraX^64^ and Fiji Image J v1.53.c^65^.

### Mouse immunisation and sampling

To prepare the RBD-SpyVLP for vaccination at 125 µg/mL (based on SpyTag-RBD concentration), 5 µM SpyTag-RBD was conjugated with 3.33 µM or 5 µM of SpyCatcher003-mi3 in TBS pH 8.0 at 4 °C for 16 h. The reaction was centrifuged for 30 min at 17,000 × *g* at 4 °C to remove potential aggregates. RBD-SpyVLP was aliquoted and stored at −80 °C. RBD-SpyVLP (125 µg/mL) or SpyTag-RBD (125 µg/mL) was diluted to 4 µg/mL (0.1 µg dose) or 20 µg/mL (0.5 µg dose) in the same buffer freshly before immunisation. An empty VLP only control (contains equivalent amount of VLP as the 0.1 µg dose of RBD-SpyVLP) diluted in the same buffer was included. Before IM immunisation, each sample was mixed 1:1 (25 µL + 25 µL) with AddaVax adjuvant (Invivogen). Mouse experiments were performed according to the UK Animals (Scientific Procedures) Act Project Licence (PBA43A2E4) and approved by the University of Oxford Local Ethical Review Body. All experiments were done in compliance with the ethical regulations for animal testing and research. Female C57BL/6 or BALB/c mice (~5 weeks old at the time of first immunisation) were obtained from BMS Oxford or Envigo. Mice were housed in accordance with the UK Home Office ethical and welfare guidelines and fed on standard chow and water ad libitum. The mouse room was kept at 18–23 °C with 45–65% humidity and 12-h light and dark cycle. Isoflurane (Abbott) lightly anaesthetised mice were immunised on day 0 and day 14 IM with 50 µL of RBD-SpyVLP at 0.1 or 0.5 µg or equivalent dose of unconjugated SpyTag-RBD or SpyCatcher003-mi3 VLP. Sera samples were obtained on day 42 via cardiac puncture of humanely sacrificed mice. The collected whole blood in microtainer SST tubes (BD) was allowed to clot at RT for 1 h before spinning down at 10,000 × *g* for 5 min. The clarified sera were heat-inactivated at 56 °C for 30 min before storing at −20 °C.

### RBD ELISA (mouse and human sera)

RBD-6H was expressed in Expi293F cells (Thermo Fisher) according to the manufacturer’s protocol and purified using HisTrap HP column (Cytivia) and desalted using Zeba Spin Desalting Column (Thermo Fisher). To detect anti-RBD antibody in the immunised mouse sera, 50 µL purified RBD-6H (amino acids 330–532) (2 µg/mL) diluted in PBS was coated on NUNC plates at 4 °C overnight. Plates were then washed with PBS and blocked with 300 μL of 5% skimmed milk in PBS for 1 h at RT. In round-bottom 96-well plates, heat-inactivated mouse sera (starting dilution 1 in 40) was diluted in PBS/0.1% BSA in a 2-fold serial dilution in duplicate. 50 μL of the diluted sera was then transferred to the NUNC plates for 1 h at RT. Plates were then washed with PBS and 50 μL of secondary HRP goat anti-mouse antibody (Dako P0447) diluted 1:800 in PBS/0.1% BSA was added to the wells for 1 h at RT. Plates were washed and developed as described above. Serum RBD-specific antibody response was expressed as EPT. EPT is defined as the reciprocal of the highest serum dilution that gives a positive signal (blank + 10SD) determined using a five-parameter logistic equation calculated using GraphPad Prism 8. RBD antibody response in the convalescent plasma was tested on a cell-based RBD ELISA, since human plasma gives high background on NUNC plates in our hands. MDCK-RBD cells were generated as previously described^26,29,66^. Briefly, MDCK-SIAT1 cells (ECACC 05071502)^67^ were stably transfected using lentiviral vector to express SARS-CoV-2 RBD (amino acid 331–529, NITN…GPKK^6^) fused at the C-terminus to the transmembrane domain of HA H7 (A/HongKong/125/2017) (EPI977395) (KLSSGYKDVILWFSFGASCFILLAIVMGLVFICVKNGNMRCTICI) for surface expression. MDCK-RBD was seeded at 3 × 10^4^ cells/well in flat-bottom 96-well plates and incubated overnight at 37 °C and 5% CO_2_ prior to the assays. Human plasma was incubated with MDCK-SIAT1 cells for 1 h at RT prior to the assays to remove background binding on MDCK-SIAT1 cells. Plates seeded with MDCK-RBD were washed with PBS and 50 μL of pre-absorbed human sera diluted in a 2-fold dilution series (starting dilution 1 in 5) were added to the cells for 1 h at RT. The same set of sera were added in parallel plates seeded with MDCK-SIAT1 to obtain background binding on MDCK-SIAT1. Plates were washed and 50 μL of a secondary Alexa Fluor 647 goat anti-human antibody (Life Technologies A21455) (1:500 in PBS with 0.1% (w/v) BSA) were added for 1 h at RT. Plates were washed and 100 μL of PBS/1% formalin was added. Fluorescence signal was then read on a Clariostar plate reader. Background signal obtained on the parallel MDCK-SIAT1 plates were subtracted. EPT was determined as described above. Convalescent plasma samples (18 mild, 10 severe/critical) were randomly selected from COVID19 samples collected from March to May 2020 at John Radcliffe Hospital, Oxford as described previously^68^. The studies of which these samples were randomly selected from were approved by the following research ethics committee: Gastro-intestinal illness in Oxford: COVID substudy [Sheffield Research Ethics Committee, reference: 16/YH/0247]ISARIC/WHO, Clinical Characterisation Protocol for Severe Emerging Infections [Oxford Research Ethics Committee C, reference 13/SC/0149], the Sepsis Immunomics project [Oxford Research Ethics Committee, reference:19/SC/0296]) and by the Scotland A Research Ethics Committee (Ref: 20/SS/0028). Written informed consent was obtained from all patients.

### Spike glycoprotein ELISA (mouse and human sera)

A cell-based ELISA as described previously^26^ was used to determine the anti-spike glycoprotein antibody response in the mouse sera and convalescent plasma. Briefly, MDCK-Spike was produced by stably transfecting parental MDCK-SIAT1 cells with full-length SARS-CoV-2 spike glycoprotein cDNA using a lentiviral vector. MDCK-Spike cells (3 × 10^4^ cells/well) were seeded in 96-well plates and incubated overnight at 37 °C. Mouse sera was diluted as above and 50 μL was transferred to the washed plates seeded with MDCK-Spike cells for 1 h at RT. Human plasma was pre-incubated with MDCK-SIAT1 cells as described above, before dilution and adding to MDCK-Spike for 1 h at RT. Parallel plates were seeded with MDCK-SIAT1 for background subtraction as described above for human plasma. For mouse sera, 50 μL of a secondary Alexa Fluor 647 goat-anti-mouse antibody (1:500) (Life Technologies A21235) was then added for 1 h at RT. For human sera, 50 μL of a secondary Alexa Fluor 647 goat-anti human antibody (1:500) (Life Technologies A21455) was used. Plates were then washed with PBS and 100 μL of PBS/1% formalin was added to each well. Fluorescence signal was read on a Clariostar plate reader and the EPT titre was calculated as described above.

### ACE2 competition ELISA

ACE2 competition ELISA was done with RBD-SpyVLP immobilised on NUNC plates as previously described with slight modifications^26^. ACE2-Fc was expressed in Expi293F cells and purified using protein A-based MabSelect SuRe column (Cytivia) and buffer-exchanged using Zeba Spin Desalting Column (Thermo Fisher) into PBS. ACE2-Fc was chemically biotinylated using EZ-Link Sulfo-NHS-LC-Biotinylation Kit (Thermo Fisher) according to manufacturer’s protocol. 25 ng/well of RBD-SpyVLP was coated on the ELISA plate at 4 °C overnight. Plates were then washed with PBS and blocked with 300 μL of 5% (w/v) skimmed milk in PBS for 1 h at RT. Heat-inactivated mouse or pig sera or human plasma samples were titrated in duplicate as half-log_10_ 8-point serial dilution, starting at 1 in 5 in 30 µL with PBS with 0.1% (w/v) BSA. 30 µL of biotinylated ACE2-Fc at 0.2 nM (40 ng/mL) (or 0.4 nM for human plasma) was added to the samples. 50 µL of the biotinylated ACE2-Fc:sample mixture was transferred to the RBD-SpyVLP-coated plates and incubated for 1 h at RT. A secondary Streptavidin-HRP (S911, Life Technologies) diluted to 1:1600 in PBS/0.1% BSA was added to the PBS-washed plates and incubated for 1 h at RT. Plates were then washed and developed as above. Biotinylated ACE2-Fc without sera or plasma was used to obtain the maximum signal and wells with PBS/BSA buffer only were used to determine the minimum signal. Graphs were plotted as % binding of biotinylated ACE2 to RBD. Binding % = (*X*−Min)/(Max−Min)*100 where *X* = measurement of the sera or plasma, Min = buffer only, Max = biotinylated ACE2-Fc alone. ACE2-blocking activity of the sera or plasma was expressed as IC_50_ determined using non-linear regression curve fit using GraphPad Prism 8.

### Authentic SARS-CoV-2 virus neutralisation assay (PRNT)

96-well plates containing a confluent monolayer of Vero-E6 cells were incubated with 10–20 plaque forming units (PFU) of SARS CoV-2 (hCoV-19/England/02/2020, EPI_ISL_407073, kindly provided by Public Health England) and two-fold serial dilution of heat-inactivated mouse sera for 3 h at 37 °C, 5% CO_2_, in triplicate per serum sample. Inoculum was then removed, and cells were overlaid with virus growth medium containing Avicel microcrystalline cellulose (final concentration of 1.2%) (Sigma-Aldrich). The plates were then incubated at 37 °C, 5% CO_2_. At 24 h post-infection, cells were fixed with 4% paraformaldehyde and permeabilised with 0.2% (v/v) Triton-X-100 in PBS, before staining to visualise virus plaques, as described previously for the neutralisation of influenza viruses^69^, but using a rabbit polyclonal to nonstructural protein 8 (NSP8) antibody (Antibodies Online; ABIN233792) and anti-rabbit-HRP conjugate (Bio-Rad) and detected using HRP on a TMB-based substrate. Virus plaques were quantified and ND_50_ for sera was calculated using LabView software as described previously^69^.

### Authentic SARS-CoV-2 plaque reduction neutralisation assay (PRNT)

SARS-CoV-2 (hCoV-19-Australia/VIC01/2020, GenBank MT007544, EPI_ISL_406844)^70^ was diluted to a concentration of around 1000 PFU/mL and 75 μL was mixed with an equal volume of minimal essential medium (MEM) (Life Technologies) containing 1% (v/v) foetal bovine serum (FBS) (Life Technologies) and 25 mM HEPES buffer (Sigma-Aldrich) with doubling pooled mouse sera dilutions (starting dilution 1:40) in a 96-well V bottomed plate. The plate was then incubated at 37 °C in a humidified incubator for 1 h before the virus–antibody mixture was transferred to 24-well plates containing confluent monolayers of Vero E6 cells (ECACC 85020206) cultured in MEM containing 10% (v/v) FBS. The plates were incubated for 1 h at 37 °C and overlaid with MEM containing 1.5% (w/v) carboxymethylcellulose (Sigma-Aldrich), 4% (v/v) FBS and 25 mM HEPES buffer. Plates were incubated at 37 °C, 5% CO_2_ for 5 days prior to fixation with 20% formalin in PBS overnight. Plates were then washed with tap water and stained with 0.2% (w/v) crystal violet solution (Sigma-Aldrich) and plaques were visualised and counted. A mid-point probit analysis (written in R programming language for statistical computing and graphics) was used to determine the dilution of antibody required to reduce numbers of SARS-CoV-2 virus plaques by 50% (ND_50_) compared with the virus-only control (*n* = 5). A human MERS convalescent serum known to neutralise SARS-CoV-2 (National Institute for Biological Standards and Control, UK) was included in each run as assay control. The RBD sequence of the hCoV-19/England/02/2020 isolate and the hCoV-19-Australia/VIC01/2020 are identical.

### mAb competition assays

mAb competition ELISA was done with RBD-SpyVLP immobilised on NUNC plates as described above with slight modifications. Plates were coated and blocked as above. Heat-inactivated mouse or pig sera or human plasma samples were titrated in duplicate as half-log 8-point serial dilution, starting at 1 in 5 in 30 µL with PBS with 0.1% (w/v) BSA or tested at 1:20 in quadruplicates. 30 µL of chemically biotinylated mAb (FI-3A, FD-11A, EY6A or S309), titrated to determine the lowest binding concentration at top plateau (all mAbs produced in house)^26,28,29^, was added to the sera. Biotinylation was conducted as described above. 50 µL of the biotinylated mAb:sera mixture was transferred to the RBD-SpyVLP coated plates and incubated for 1 h at RT. A secondary Streptavidin-HRP (Life Technologies, S911) diluted to 1:1600 in PBS/0.1% BSA was added to the PBS-washed plates and incubated for 1 h at RT. Plates were then washed and developed as above. Biotinylated mAb without sera or plasma was used to obtain the maximum signal and wells with PBS with 0.1% (w/v) BSA buffer only were used to determine the minimum signal. Graphs were plotted as % binding of biotinylated mAb to RBD-SpyVLP. Binding % = (*X*−Min)/(Max−Min)*100, where *X* = measurement of the sera or plasma, Min = buffer only, Max = biotinylated mAb alone.

### Pig immunisation and sampling

Pig studies were performed in accordance with the UK Animals (Scientific Procedures) Act 1986 and with approval from The Pirbright Institute Local Animal Welfare and Ethical Review Body (AWERB) (Project Licence PP1804248). RBD-SpyVLP for pig immunisation was prepared as above. Nine weaned, Large White-Landrace–Hampshire cross-bred pigs of 8–10 weeks of age from a commercial rearing unit were randomly allocated to three treatment groups (5 µg RBD-SpyVLP, 50 µg RBD-SpyVLP or 100 µg spike glycoprotein) (*n* = 3). RBD-SpyVLP was diluted to 5 or 50 µg/mL in 25 mM Tris–HCl, pH 8.0, 150 mM NaCl or spike glycoprotein diluted to 100 µg/mL in PBS and mixed with an equal amount of AddaVax (Invivogen) (1 mL + 1 mL) prior to immunisation. The spike glycoprotein was a soluble trimeric spike with the pre-fusion stabilisation substitutions (K983P, V984P, furin cleavage site removed and inclusion of a C-terminal T4-foldon domain for trimerization)^71^. The spike glycoprotein was expressed in Expi293F cells and purified as previously described^36^. Briefly, supernatant containing soluble spike glycoprotein was purified using immobilised metal affinity followed by gel filtration in TBS (pH 7.4). Pigs were dosed via IM injection into the brachiocephalic muscle with 2 mL of RBD-SpyVLP (5 or 50 µg) or spike (100 µg) at day 0 and day 28. Blood samples were taken on a weekly basis at 0, 7, 14, 21, 28, 35, 42 and 56 days post-immunisation (DPI) by venepuncture of the external jugular vein: 8 mL/pig in BD SST vacutainer tubes (Fisher Scientific) for serum collection and 40 mL/pig in BD heparin vacutainer tubes (Fisher Scientific) for PBMC isolation. Additional heparin blood samples were collected on 31 and 33 DPI to track the plasma cell response to boost. Sera samples were stored at −20 °C and heat-inactivated at 56 °C for 2 h before use in pVNT or VNT assays. Oral and nasal swabs were collected weekly and placed in 500 µL Media 199 (Thermo Fisher) supplemented with 0.0025% (w/v) Nystatin (Merck), 0.01% penicillin–streptomycin (Gibco), 0.025% (w/v) 1 M HEPES solution (Gibco), 0.005% (w/v) sodium bicarbonate (Merck) and 0.067% (w/v) BSA (Merck) (VTM). Swabs were centrifuged at 700 × *g* for 5 min before aspirating the liquid and storing with the swab at −20 °C. Prior to assessment of antibodies, swabs and VTM were loaded in Spin-X centrifuge 0.45 µm columns (Fisher Scientific) and fluid collected by centrifugation at 21,000 × *g* for 5 min.

### RBD ELISA (pig sera and swab fluids)

An ELISA to analyse anti-RBD antibody response in pig sera was performed as previously described^36^. Briefly, 50 µL of 2 µg/mL purified RBD-6H as described above was coated on flat-bottomed 96-well plates (Immunon 4 HBX; Thermo Fisher Scientific) overnight at 4 °C. Plates were washed with TBS (pH 7.4) with 0.1% (v/v) Tween-20 and blocked with 100 µL of PBS containing 3% (w/v) skimmed milk for 1 h at RT. Pig sera samples were diluted in PBS with 1% (w/v) skimmed milk and 0.1% (v/v) Tween-20 in a 2-fold serial dilution starting at 1:10 dilution and 100 µL of the diluted sera was added to the coated plates for 1 h at RT. A conjugated secondary goat anti-pig IgG HRP (Abcam, Cambridge, UK) at 1:10,000 dilution in PBS with 1% (w/v) skimmed milk and 0.1% (v/v) Tween-20 was added for 1 h at RT. Plates were washed and 100 µL TMB (One Component Horse Radish Peroxidase Microwell Substrate, BioFX, Cambridge Bioscience) was added to each well and the plates were incubated for 7 min at RT. 100 µL BioFX 450 nm Stop Reagent (Cambridge Bioscience) was then added and absorbance was determined using a microplate reader. End-point antibody titres (mean of duplicates) were defined as following: the log_10_ OD was plotted against the log_10_ sample dilution and a regression analysis of the linear part of this curve allowed calculation of the EPT with an absorbance of twice the mean absorbance of pre-immunised sera. RBD-specific antibody titres in oral and nasal swab fluids were determined by ELISA as detailed above except that the conjugated secondary antibody was replaced with either goat anti-porcine IgG HRP (Bio-Rad Antibodies) at 1:20,000 dilution in PBS with 1% (w/v) skimmed milk and 0.1% (v/v) Tween-20 or goat anti-porcine IgA HRP (Bio-Rad Antibodies) at 1:20,000 dilution in the same diluent.

### Virus neutralisation assay (VNT) (pig sera)

VNT on pig sera was done as described previously^36^. Briefly, Vero E6 cells were seeded in 96-well flat-bottom plates (1 × 10^5^ cells/mL) and incubated at 37 °C overnight prior to the assays. Two-fold serial dilutions of sera (starting dilution 1 in 5) in quadruplicate were prepared in 96-well round-bottom plates using Dulbecco’s modified Eagle medium (DMEM) with 1% (v/v) FBS and 1% antibiotic–antimycotic (Gibco). 75 µL of the diluted sera was mixed with an equal volume of media containing 64 PFU of SARS-CoV-2 virus (hCoV-19/England/02/2020, EPI_ISL407073) and incubated for 1 h at 37 °C. Media in the wells seeded with Vero E6 was replaced with 100 µL DMEM with 10% (v/v) FBS and 1% antibiotic–antimycotic (Gibco) and 100 µL of the sera–virus mixture was added into the wells. The plates were incubated for 6 days at 37 °C. Cytopathic effect (CPE) was monitored on a brightfield microscopy, and by fixation using formaldehyde (VWR) and staining using 0.1% (w/v) Toluidine Blue (Sigma-Aldrich). CPE was scored by researchers who were blinded to the identity of the samples. No sera or no virus controls were run in parallel on each plate. Neutralisation titres (ND5_0_) were expressed as the reciprocal of the serum dilution that prevented CPE in 50% of the wells.

### Pseudovirus neutralisation test (pVNT) (pig sera)

Lentiviral-based SARS-CoV-2 pseudoviruses were generated as described previously^36^. Briefly, HEK293T cells were seeded at a density of 7.5 × 10^5^ in six-well plates before transfection with the following plasmids: 500 ng of SARS-CoV-2 spike, 600 ng p8.91 (HIV-1 gag-pol), 600 ng CSFLW (lentiviral genome plasmid encoding a firefly luciferase transgene)^36^ using 10 µL polyethylene imine (PEI) (1 µg/mL) in Opti-MEM media (Thermo Fisher). The media was replaced with 3 mL DMEM with 10% (v/v) FBS and incubated at 37 °C. Supernatant containing pseudovirus was harvested at 48 and 72 h post transfection. Collected supernatant was centrifuged at 1300 × *g* for 10 min at 4 °C to remove debris. To perform the assay, 2 × 10^4^ HEK293T target cells transfected with 500 ng of a human ACE2 expression plasmid (Addgene 1786) were seeded in a white flat-bottom 96-well plate one day prior to the assays. Pig sera were diluted with a four-fold serial dilution in Opti-MEM media (starting dilution 1 in 20) and 50 µL was added to a 96-well plate in quadruplicate. Pseudovirus pre-titrated to give 1 × 10^6^ relative light unit (RLU) in 50 µL DMEM with 10% (v/v) FBS was added to the sera and incubated at 37 °C for 1 h. The pseudovirus:sera mix was then transferred to the target cells and incubated at 37 °C for 72 h. Firefly luciferase activity was measured using BrightGlo luciferase reagent on a GloMax-Multi+ Detection System (Promega). Pseudovirus neutralisation titres (ND_50_) were expressed as the reciprocal of the serum dilution that inhibited luciferase signal in 50% of the wells.

### Intracellular cytokine staining assay (pig PBMC)

Assessment of intracellular cytokine expression following stimulation of PBMCs with synthetic peptides representing SARS-CoV-2 S protein was conducted as described previously^36^. In brief, PBMCs were isolated from heparinized blood by density gradient centrifugation and suspended at 1 × 10^7^ cells/mL in RPMI-1640 medium, GlutaMAX supplement, HEPES (Gibco) supplemented with 10% (v/v) heat-inactivated FBS (New Zealand origin, Life Science Production), 1% penicillin–streptomycin and 0.1% (v/v) 2-mercaptoethanol (50 mM; Gibco) (cRPMI). 50 µL PBMC were added per well to 96-well round bottom plates and stimulated in triplicate wells with SARS-CoV-2 S peptide pools at a final concentration of 1 µg/mL peptide. Unstimulated cells in triplicate wells were used as a negative control. After 14 h incubation at 37 °C, 5% CO_2_, cytokine secretion was blocked by addition of 1:1000 BD GolgiPlug (BD Biosciences) and cells were further incubated for 6 h at 37 °C. PBMC were surface-labelled with Zombie NIR fixable viability stain (BioLegend), CD3-FITC mAb (clone BB23-8E6-8C8, BD Biosciences), CD4-PerCP-Cy5.5 mAb (clone 74-12-4, BD Bioscience) and CD8α-PE mAb (clone 76-2-11, BD Bioscience). After fixation (Fixation Buffer, BioLegend) and permeabilization (Permeabilization Wash Buffer, BioLegend), cells were stained with: IFN-γ-Alexa Fluor 647 mAb (clone CC302, Bio-Rad Antibodies,) and TNF-α-Brilliant Violet 421 mAb (clone Mab11, BioLegend). Cells were analysed using a BD LSRFortessa flow cytometer (BD Biosciences) and data analysed using FlowJo software (BD Biosciences). Total SARS-CoV-2 S-specific IFN-γ-positive responses for live CD3^+^CD4^+^ and CD3^+^CD4^-^CD8^+^ T cells are presented after subtraction of the background response detected in the media-stimulated control PBMC samples of each pig, prior to summing together the frequency of S-peptide pools 1–3 specific cells (see Fig. S5c for representative flow cytometry plots).

### RBD-tetramer staining assay (pig PBMC)

A biotinylated form of RBD was generated for B-cell tetramer staining assays. An RBD protein with a C-terminal biotin acceptor peptide (RBD-BAP) was expressed from plasmid pOPINTTGNeo in Expi293F cells according to the manufacturer’s instructions. Culture supernatants were clarified by centrifugation and purified through a 5 mL HisTrap FF column (GE Healthcare), using the ÄKTA Pure chromatography system (Cytiva). Fractions containing RBD-BAP were concentrated and the excess imidazole removed by buffer exchange using an Amicon 10 kDa (Merck). RBD-BAP was biotinylated using *E. coli* biotin ligase (BirA). GST-BirA enzyme was expressed, purified and biotinylated as previously described^72^. Biotinylation reactions were assembled with 100 µM RBD-BAP, 1 µM GST-BirA, 5 mM magnesium chloride (Ambion), 2 mM ATP and 150 µM d-biotin (both Merck) and incubated twice for one hour at 30 °C with additional fresh biotin and GST-BirA added in between incubations. GST-BirA was removed from the reaction with a GST HiTrap column, as above, and RBD-BAP was purified by dialysis as above. Biotinylation of RBD-BAP was confirmed by streptavidin band shift assay^72^ and quantified by BCA assay (Pierce). RBD tetramers were assembled by combining biotinylated RBD with streptavidin-Brilliant Violet 421 or streptavidin-Brilliant Violet 650 (both BioLegend) at a molar ratio of 4:1. Negative control ‘decoy’ tetramers were similarly assembled using biotinylated Nipah virus soluble glycoprotein^73^ and streptavidin-PerCP Cy5.5 (BioLegend).

PBMC were stained with SARS-CoV-2 S RBD-tetramers to assess the frequency of circulating specific B cells during the course of the study. For 28, 31, 33 and 35 days post-infection, fresh PBMC were analysed (in triplicate), while for 0, 7, 14, 42 and 56 days post-infection, previously cryopreserved PBMC were assessed (in quadruplicate). RBD tetramers were assembled by combining biotinylated RBD with streptavidin-Brilliant Violet 421 or streptavidin-Brilliant Violet 650 (both BioLegend) at a molar ratio of 4:1. Negative control ‘decoy’ tetramers were similarly assembled using biotinylated Nipah virus soluble glycoprotein^73^ and streptavidin-PerCP Cy5.5 (BioLegend). PBMC were washed in cold PBS and seeded at 1 × 10^6^ cells/well in 96-well round bottom plates and with combinations of RBD and decoy tetramers by incubation for 30 min on ice. After washing, cells were stained with Zombie Aqua, CD3-PE-Cy7 mAb (clone BB23-8E6-8C8, BD Biosciences), CD14-PE Vio 770 mAb (clone REA599, Miltenyi Biotec), IgG-Alexa Fluor-647 mAb (Cohesion Biosciences, Generon,), IgA-FITC polyclonal Ab (BioRad Antibodies) and IgM-PE mAb (clone K52 1C3, BioRad Antibodies; conjugated using Lightning-Link^®^ PE Antibody Labeling Kit, Expedeon, Abcam), for 30 min on ice. After washing and fixation in 4% paraformaldehyde for 30 min at 4 °C, cells were analysed on a BD LSRFortessa flow cytometer with downstream analysis using FlowJo software. Following exclusion of dead cells, CD3^+^, CD14^+^ and decoy tetramer+ cells, the percentage of IgA^+^, IgG^+^ or IgM^+^ cells dual-labelled with both RBD tetramers was assessed (see Fig. S4c for representative flow cytometry plots).

### RBD IgG ELISpot assay (pig PBMC)

Sterile 96-well Multiscreen-HA filter plates with a mixed cellulose membrane (MAHAS4510, Millipore) were coated with 100 μL of 15 μg/mL mouse anti-porcine IgG mAb (clone MT421, Mabtech, 2BScientific) diluted in 0.05 M carbonate–bicarbonate buffer pH 9.2 (Merck). Coated plates were incubated for a minimum of 18 h at 4 °C. Plates were then washed with PBS and blocked using cRPMI for at least 1 h at 37 °C, 5% CO_2_, 95% humidity. Blocking solution was then removed, and PBMC were added at a density of 5 × 10^5^/well for antigen-specific response or at 5 × 10^4^/well for wells assigned to total IgG (positive control). The plates were then incubated for 18 h at 37 °C, 5% CO_2_, 95% humidity. Media was removed and cells were lysed with cold distilled water, followed by three PBS washes as before. To measure total IgG, 50 μL/well of biotinylated anti-IgG mAb (clone MT424-biotin, Mabtech) was added at 0.5 µg/mL. To assess antigen-specific responses 50 μL/well of biotinylated SARS-CoV-2 RBD was added at 2.5 μg/mL. As a negative control, 50 μL/well of biotinylated Nipah G protein was added to the relevant wells at 2.5 μg/mL. All antigens were diluted in PBS with 0.5% (v/v) FCS. An additional set of negative control wells were also prepared by adding 50 μL/well PBS with 0.5% (v/v) FCS. Each condition was tested in triplicate. Plates were incubated for 2 h at RT, before washing five times with PBS. Following this, 50 μL/well of streptavidin–alkaline phosphatase (streptavidin–ALP) enzyme conjugate (Mabtech) (diluted 1:1000 in PBS with 0.5% (v/v) FCS) was added to each well and plates were incubated for 1 h at RT (protected from light). Streptavidin–ALP was removed, and plates were washed another five times with PBS, followed by addition of 50 μL/well BCIP/NBTplus substrate (Mabtech), neat. Plates were left for 30 min, until distinct spots developed. Finally, development was stopped by addition of 150 μL/well of 4 °C distilled water followed by rinsing both the front and back of the plates with copious tap water. Plates were air-dried, before spots were counted using a CTL ImmunoSpot Analyzer (Cellular Technologies).

### Statistical analysis

All statistical analyses were performed using GraphPad Prism 8 (GraphPad Software). Statistical differences were analysed using either two-tailed Mann–Whitney *U* test or Kruskal–Wallis test followed by Dunn’s multiple comparisons. A *p* value < 0.05 was deemed statistically significant.

### Reporting summary

Further information on research design is available in the Nature Research Reporting Summary linked to this article.

## Supplementary information

Supplementary Information

Reporting Summary

## Data Availability

The datasets generated during and/or analysed during the current study are available from the corresponding author on reasonable request. The assession numbers to the structures of antibodies, nanobodies, SpyTag/SpyCatcher or the mi3 particles referred in this paper are obtained from publicly available sources as following: PDB 4mli, PDB 6yz5, PDB 5kp9, PDB 6W41, PDB 6WPT and PDB 6M0J. EM maps and models of the RBD_SpyVLP generated from this study are deposited in the EMDB and wwPDB under accession codes EMD-11997 and PDB 7B3Y (RBD-SpyVLP). Source data are provided with this paper.

## Source data

Source Data
