# Supplementary material for: A COVID-19 vaccine candidate using SpyCatcher multimerization of the SARS-CoV-2 spike protein receptor-binding domain induces potent neutralising antibody responses
Source: Nat Commun. 2021 Jan 22;12:542. doi: 10.1038/s41467-020-20654-7 (PMC7822889; doi:10.1038/s41467-020-20654-7)
Supplement: Supplementary file 1 — Supplementary Information [file 41467_2020_20654_MOESM1_ESM.pdf]

A COVID-19 vaccine candidate using SpyCatcher multimerization of the SARS-CoV-2 spike protein receptor-binding domain induces potent neutralising antibody responses

AUTHORS

Tiong Kit Tan[1*†], Pramila Rijal[1,2*], Rolle Rahikainen[3], Anthony H. Keeble[3], Lisa Schimanski[1,2], Saira Hussain[4], Ruth Harvey[4], Jack W.P. Hayes[5], Jane C. Edwards[5], Rebecca K. McLean[5], Veronica Martini[5], Miriam Pedrera[5], Nazia Thakur[5], Carina Conceicao[5], Isabelle Dietrich[5], Holly Shelton[5], Anna Ludi[5], Ginette Wilsden[5], Clare Browning[5], Adrian K. Zagrajek[5], Dagmara Bialy[5], Sushant Bhat[5], Phoebe Stevenson-Leggett[5], Philippa Hollinghurst[5,6], Matthew Tully[5], Katy Moffat[5], Chris Chiu[5], Ryan Waters[5], Ashley Gray[5], Mehreen Azhar[5], Valerie Mioulet[5], Joseph Newman[5], Amin S. Asfor[5], Alison Burman[5], Sylvia Crossley[5], John A. Hammond[5], Elma Tchilian[5], Bryan Charleston[5], Dalan Bailey[5], Tobias J. Tuthill[5], Simon P. Graham[5], Helen M. E. Duyvesteyn[7], Tomas Malinauskas[7], Jiandong Huo[7,8], Julia A. Tree[9], Karen R. Buttigieg[9], Raymond J. Owens[7,8], Miles W. Carroll[9,10], Rodney S. Daniels[4], John W. McCauley[4], David I. Stuart[2,7,13], Kuan-Ying A. Huang[11,12], Mark Howarth[3†], Alain R. Townsend[1,2†]

[1]MRC Human Immunology Unit, MRC Weatherall Institute of Molecular Medicine, John Radcliffe Hospital, University of Oxford, Oxford OX3 9DS, UK

[2]Centre for Translational Immunology, Chinese Academy of Medical Sciences Oxford Institute, University of Oxford, Oxford, UK

[3]Department of Biochemistry, University of Oxford, South Parks Road, Oxford OX1 3QU, UK

[4]Worldwide Influenza Centre, The Francis Crick Institute, 1 Midland Road, London NW1 1AT, UK

[5]The Pirbright Institute, Ash Road, Pirbright GU24 0NF, UK

[6]Department of Microbial Sciences, Faculty of Health and Medical Sciences, University of Surrey, Guildford, GU2 7XH, UK

[7]Division of Structural Biology, Nuffield Department of Medicine, University of Oxford, The Wellcome Centre for Human Genetics, Headington, Oxford, UK.
[8]Protein Production UK, Research Complex at Harwell, and Rosalind Franklin Institute, Rutherford Appleton Laboratory, Harwell, Didcot, OX11 0FA, UK
[9]National Infection Service, Public Health England, Porton Down, Salisbury, SP4 0JG, UK.
[10]Nuffield Department of Medicine, Wellcome Trust Centre for Human Genetics, University of Oxford, Oxford, OX3 7BN, UK
[11]Research Center for Emerging Viral Infections, College of Medicine, Chang Gung University, Taoyuan, Taiwan
[12]Division of Pediatric Infectious Diseases, Department of Pediatrics, Chang Gung Memorial Hospital, Taoyuan, Taiwan
[13]Diamond Light Source Ltd, Harwell Science & Innovation Campus, Didcot, UK

*T.K.T. and P.R. contributed equally

†Correspondence to Tiong Kit Tan (tiong.tan@rdm.ox.ac.uk), Mark Howarth (mark.howarth@bioch.ox.ac.uk) and Alain Townsend (alain.townsend@imm.ox.ac.uk)

Supplementary Information

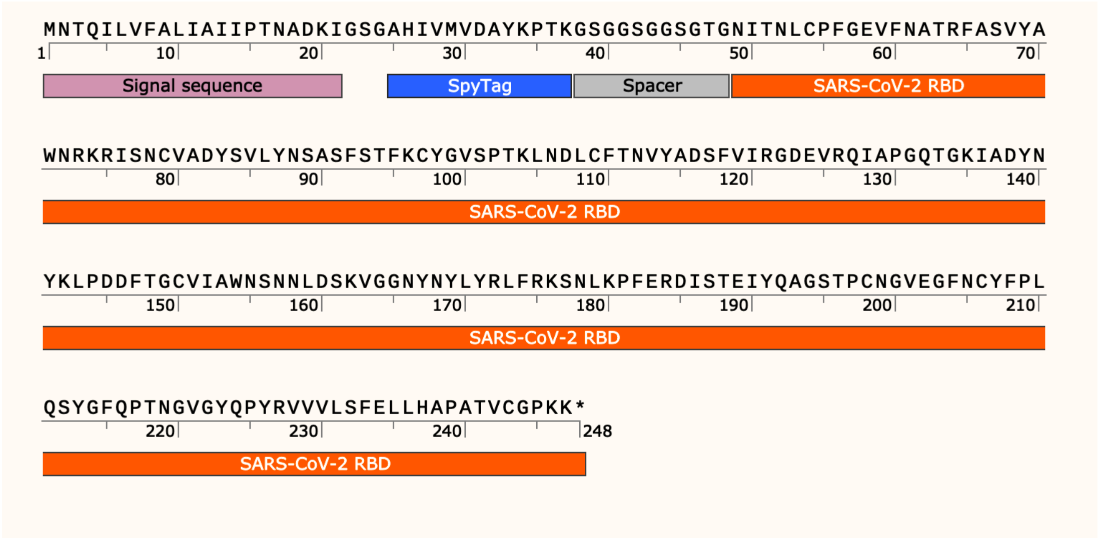

Figure S1: SpyTag-RBD expression construct. The expression cassette of SpyTag-RBD comprises the signal sequence from influenza H7 haemagglutinin (A/HongKong/125/2017) (mauve), SpyTag (blue), spacer (grey) and SARS-CoV-2 RBD (amino acid 331-529, NITN…GPKK[1], orange). Diagram adopted from SnapGene.

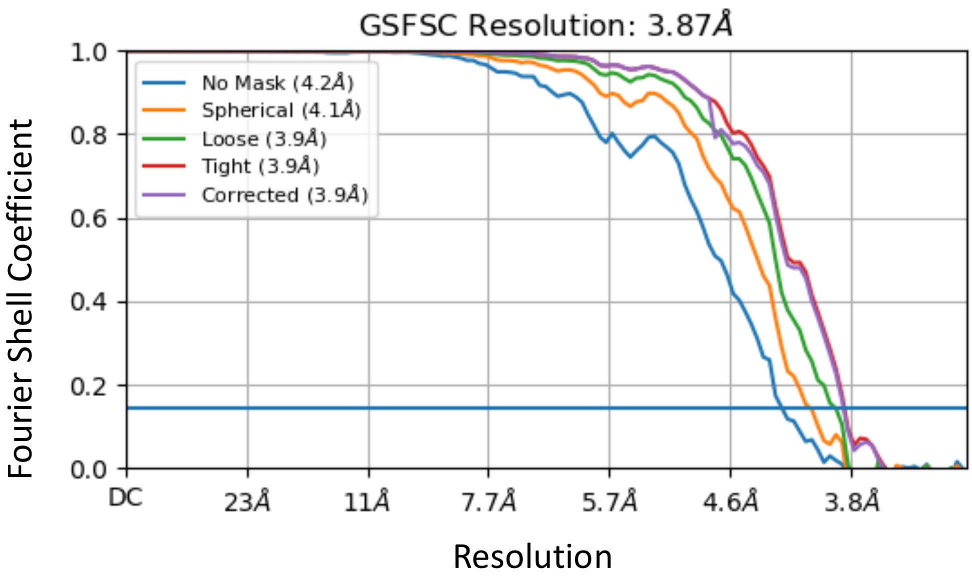

Figure S2. Gold-standard Fourier Shell Coefficient (FSC) curve following non-uniform refinement with I1 symmetry imposed. The blue horizontal line shows the FSC at 0.143. DC= Direct Current.

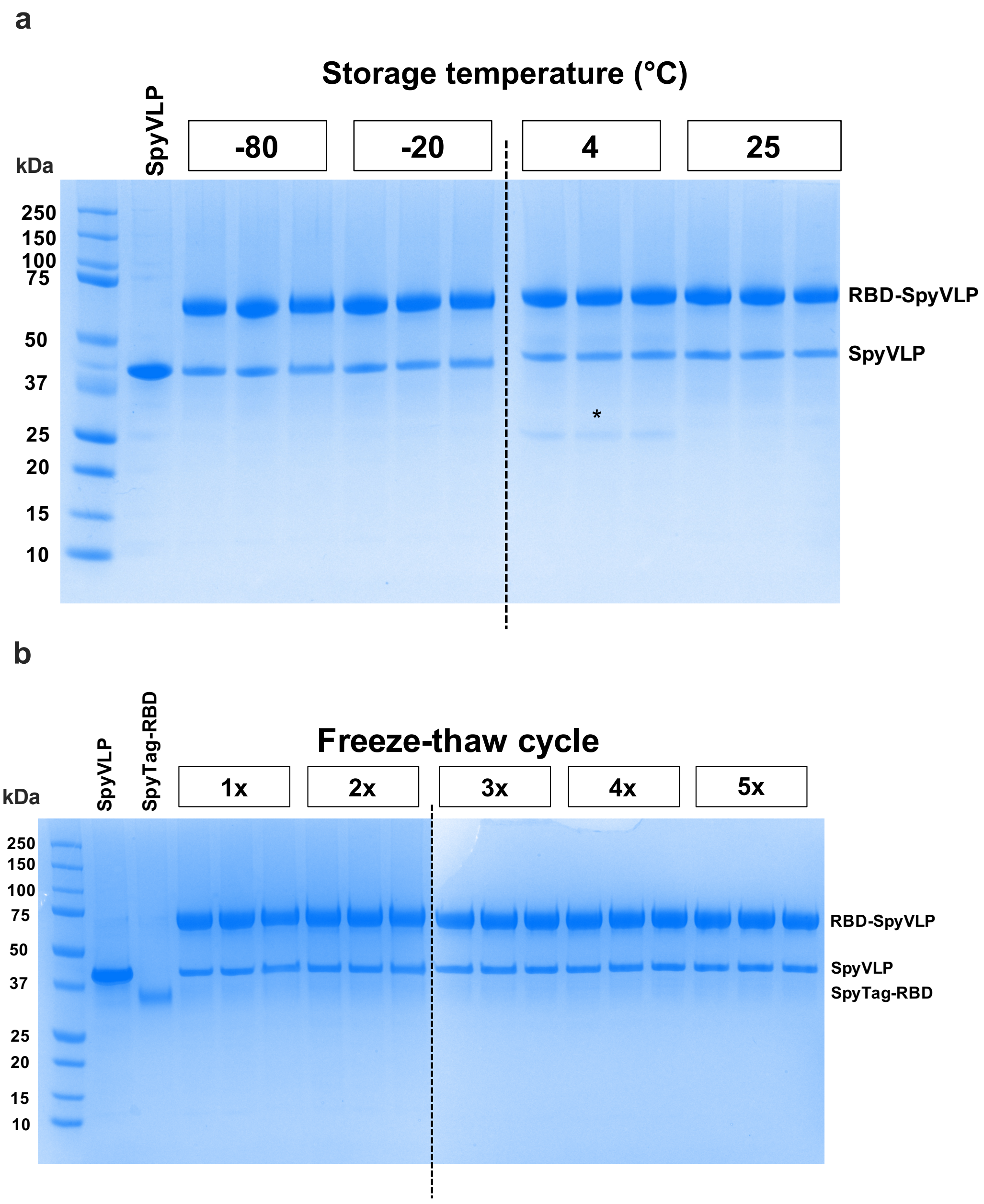

Figure S3: Analysis of the thermostability of RBD-SpyVLPs. (a) RBD-SpyVLPs (n=3 for each temperature) were stored at the indicated temperatures for two weeks and insoluble material was removed by centrifugation prior to analysis on SDS-PAGE with Coomassie staining. Solubility was calculated using densitometry and presented in Figure 2d. (b) RBD-SpyVLPs (n=15, n=3 per condition) were subjected to 1-5 rounds of free-thaw cycles as indicated, insoluble material was removed by centrifugation and analysed on SDS-PAGE with Coomassie staining. Solubility was calculated using densitometry and presented in Figure 2b. * trace of proteolysis product.

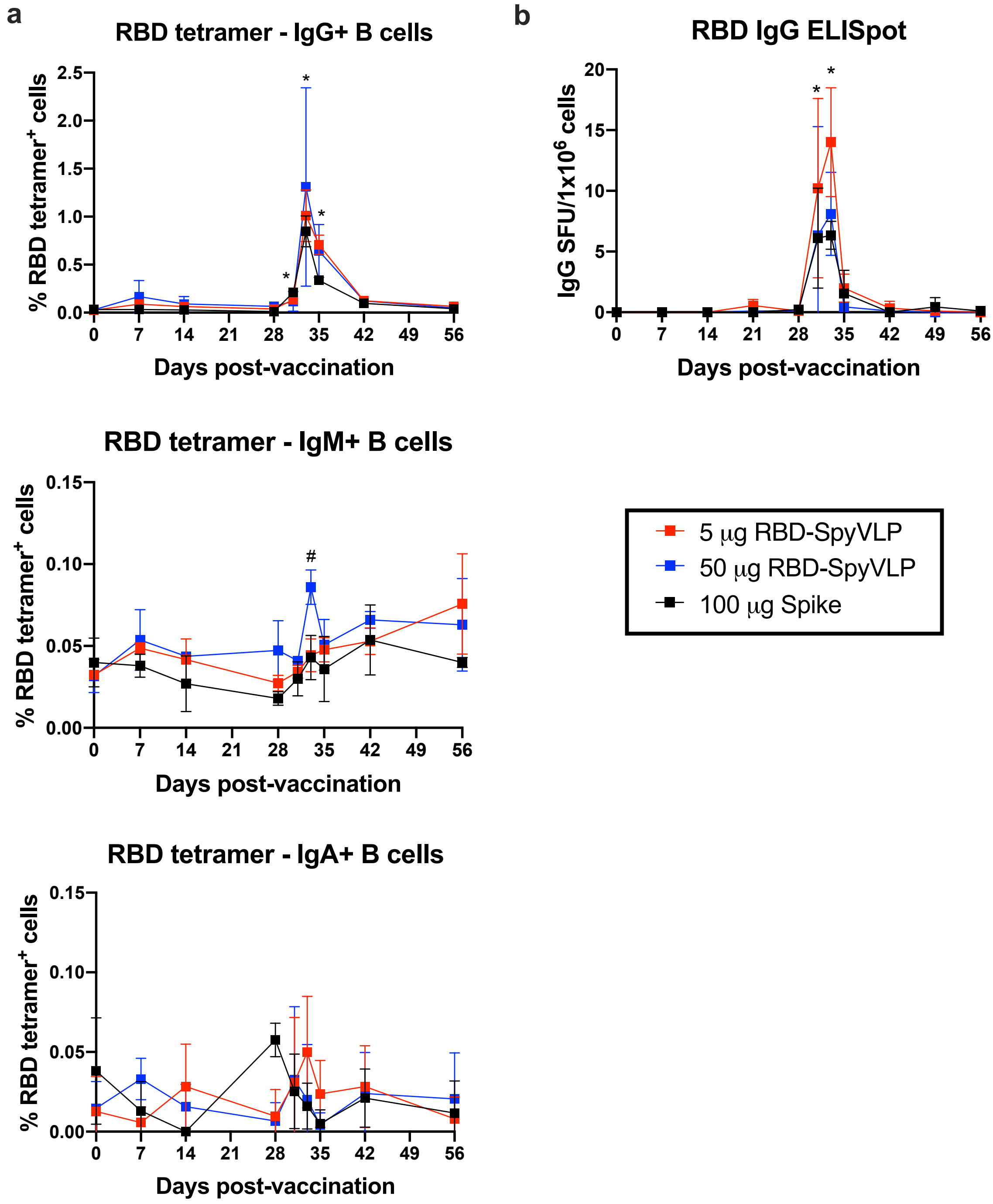
a
RBD tetramer - IgG+ B cells
% RBD tetramer+ cells
Days post-vaccination
b
RBD IgG ELISpot
IgG SFU/1x10^6 cells
Days post-vaccination
RBD tetramer - IgM+ B cells
% RBD tetramer+ cells
Days post-vaccination
RBD tetramer - IgA+ B cells
% RBD tetramer+ cells
Days post-vaccination
5 μg RBD-SpyVLP
50 μg RBD-SpyVLP
100 μg Spike

c

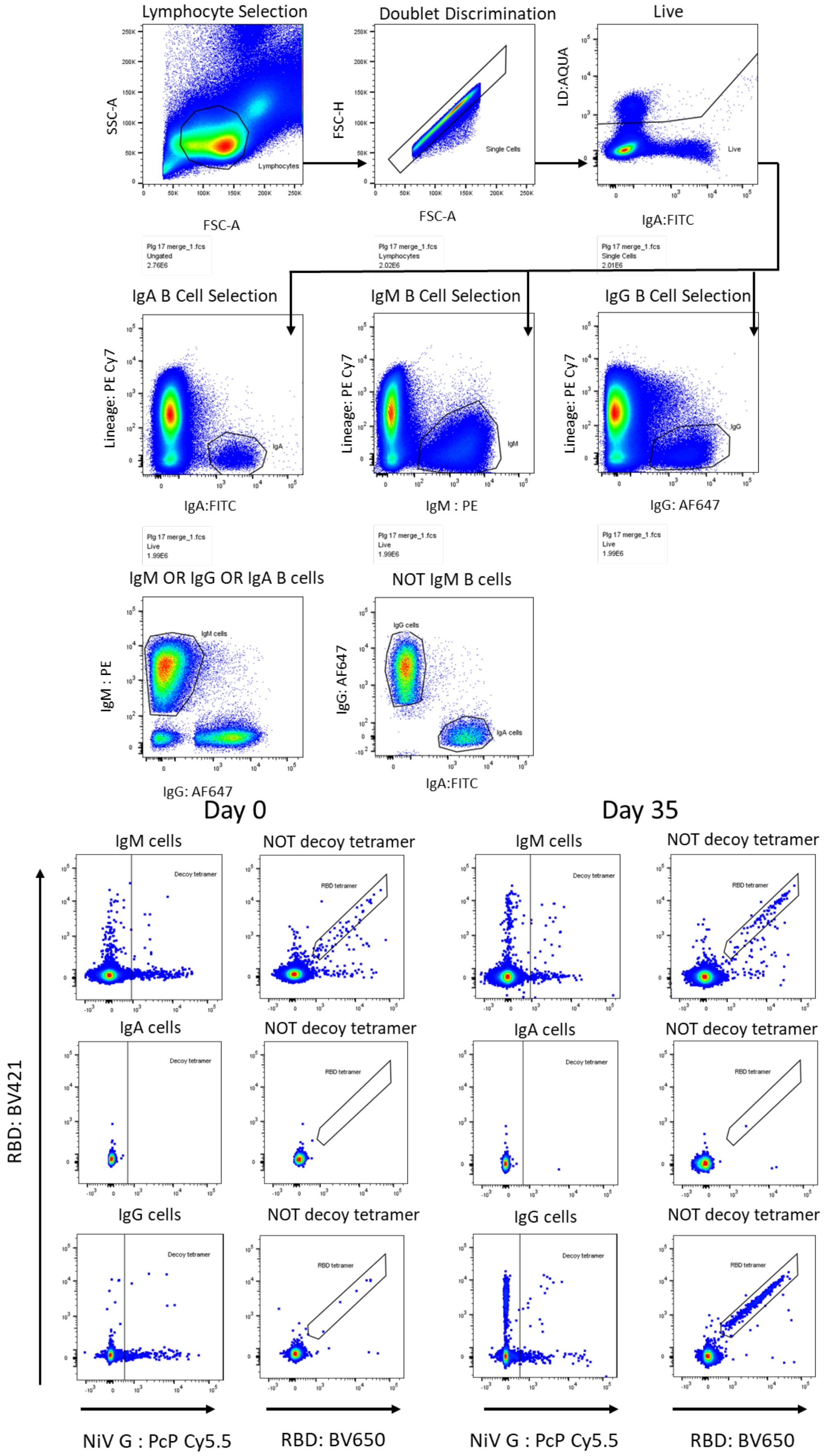

Lymphocyte Selection
Doublet Discrimination
Live
SSC-A
FSC-A
FSC-H
FSC-A
LD:AQUA
IgA:FITC
Lymphocytes
Single Cells
Live
Pig 17 merge_1.fcs
Ungated
2.76E6
Pig 17 merge_1.fcs
Lymphocytes
2.02E6
Pig 17 merge_1.fcs
Single Cells
2.01E6
IgA B Cell Selection
IgM B Cell Selection
IgG B Cell Selection
Lineage: PE Cy7
IgA:FITC
IgM : PE
IgG: AF647
IgA
IgM
IgG
Pig 17 merge_1.fcs
Live
1.99E6
IgM OR IgG OR IgA B cells
NOT IgM B cells
IgM : PE
IgG: AF647
IgM cells
IgG cells
IgA cells
IgG: AF647
IgA:FITC
Day 0
Day 35
IgM cells
NOT decoy tetramer
IgA cells
IgG cells
Decoy tetramer
RBD tetramer
RBD: BV421
NiV G : PcP Cy5.5
RBD: BV650

Figure S4: B cell analyses of pigs immunised with RBD-SpyVLPs or Spike glycoprotein. SARS-CoV-2 S-specific B cell responses were longitudinally assessed in PBMC following immunisation of pigs with RBD-SpyVLP (5 µg or 50 µg) or 100 µg Spike glycoprotein on 0- and 28-days post-immunisation. (a) B cell responses were assessed by flow cytometric analysis of IgG^+, IgM^+ and IgA^+ cells binding to RBD-tetramers, data shown as group mean ± 1SEM (n=3 biological independent samples). (b) RBD-specific plasma cell responses were assessed by IgG ELISpot assay. SFU = spot-forming units. Data points in A and B represent the mean ± 1 SEM (n=3 biological independent samples). (c) Illustration of gating strategy to assess SARS-CoV-2 S RBD tetramer staining of porcine B cells. In brief, IgM^+, IgA^+ and IgG^+ B cells were gated from live, singlet, 'lineage' negative cells. B cells stained with a NiV G decoy tetramer were excluded before assessment of dual staining with RBD tetramers (conjugated to BV421 or BV650). Representative data shown are from Pig 17 (50 µg RBD-SpyVLP) on day 0 and 35. *$p<0.05$, two-tailed T-test compared to pre-immunisation samples. #$p<0.05$, one-way ANOVA test followed by Tukey's posthoc test to compare between groups at the indicated timepoints.

a

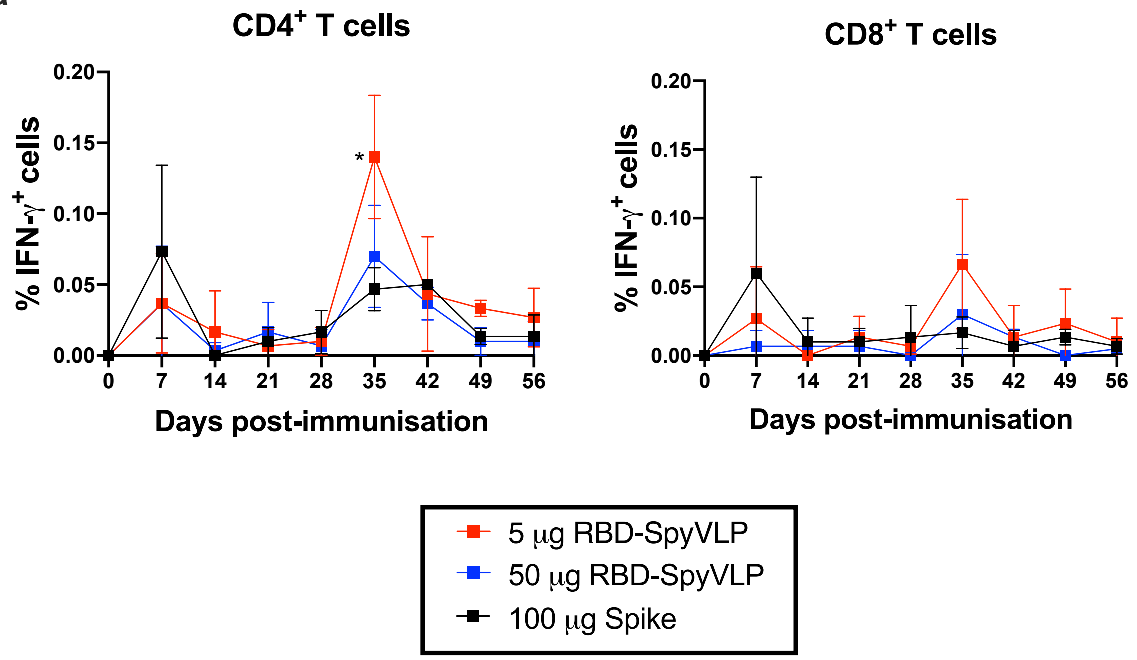

CD4+ T cells
% IFN-γ+ cells
0.20
0.15
0.10
0.05
0.00
*
0
7
14
21
28
35
42
49
56
Days post-immunisation
CD8+ T cells
% IFN-γ+ cells
0.20
0.15
0.10
0.05
0.00
0
7
14
21
28
35
42
49
56
Days post-immunisation
5 μg RBD-SpyVLP
50 μg RBD-SpyVLP
100 μg Spike

b

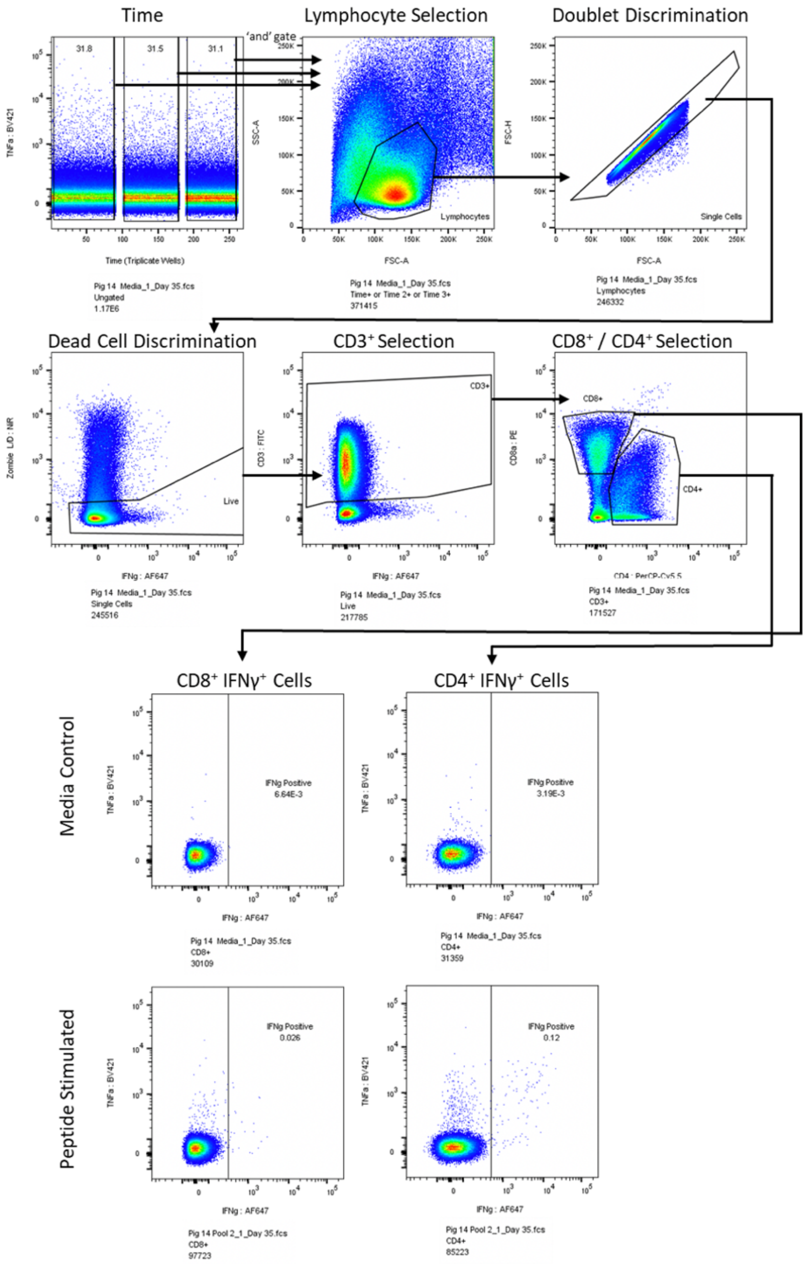
Time
Lymphocyte Selection
Doublet Discrimination
'and' gate
31.8
31.5
31.1
TNFa : BV421
Time (Triplicate Wells)
Pig 14 Media_1_Day 35.fcs
Ungated
1.17E6
SSC-A
FSC-A
Lymphocytes
Pig 14 Media_1_Day 35.fcs
Time+ or Time 2+ or Time 3+
371415
FSC-H
Single Cells
Pig 14 Media_1_Day 35.fcs
Lymphocytes
246332
Dead Cell Discrimination
CD3+ Selection
CD8+ / CD4+ Selection
Zombie L/D : NIR
IFNg : AF647
Live
Pig 14 Media_1_Day 35.fcs
Single Cells
245516
CD3 : FITC
CD3+
Pig 14 Media_1_Day 35.fcs
Live
217785
CD8a : PE
CD8+
CD4+
CD4 : PerCP-Cy5.5
Pig 14 Media_1_Day 35.fcs
CD3+
171527
CD8+ IFNγ+ Cells
CD4+ IFNγ+ Cells
Media Control
IFNg Positive
6.64E-3
Pig 14 Media_1_Day 35.fcs
CD8+
30109
IFNg Positive
3.19E-3
Pig 14 Media_1_Day 35.fcs
CD4+
31359
Peptide Stimulated
IFNg Positive
0.026
Pig 14 Pool 2_1_Day 35.fcs
CD8+
97723
IFNg Positive
0.12
Pig 14 Pool 2_1_Day 35.fcs
CD4+
85223

c

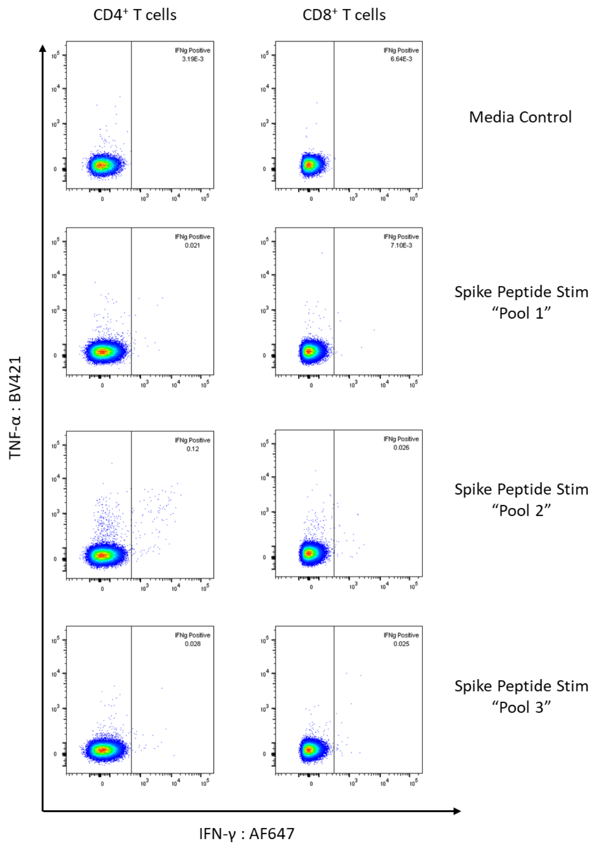

Figure S5: T cell responses of pigs immunised with RBD-SpyVLPs or Spike glycoprotein. SARS-CoV-2 S-specific T cell responses were longitudinally assessed in PBMCs following immunisation of pigs with RBD-SpyVLP (5 µg or 50 µg) or 100 µg Spike glycoprotein on 0- and 28-days post-immunisation. (a) T cell responses were assessed by flow cytometry following stimulation with S peptides. IFN-γ expression by $CD4^+$ and $CD8^+$ T cells was determined by intracellular cytokine staining. Data points represent the mean ± 1 SEM (n=3 biological independent samples). (b) Illustration of gating strategy to assess cytokine responses of SARS-CoV-2 S peptide stimulated porcine PBMCs. In brief, $CD3^+CD8^+$ and $CD3^+CD4^+$ T cells were gated from live, singlet cells and assessed for their expression of IFN-γ. Representative data shown are from Pig 17 (50 µg RBD-SpyVLP) 0 and 35 days post-immunisation. (c) Illustration of cytokine responses of porcine PBMCs to mock and spike peptide pool stimulation. Representative data shown are from Pig 14 (5 µg RBD-SpyVLP) 35 days post-immunisation. * $p<0.05$, two-tailed T-test compared to pre-immunisation samples.

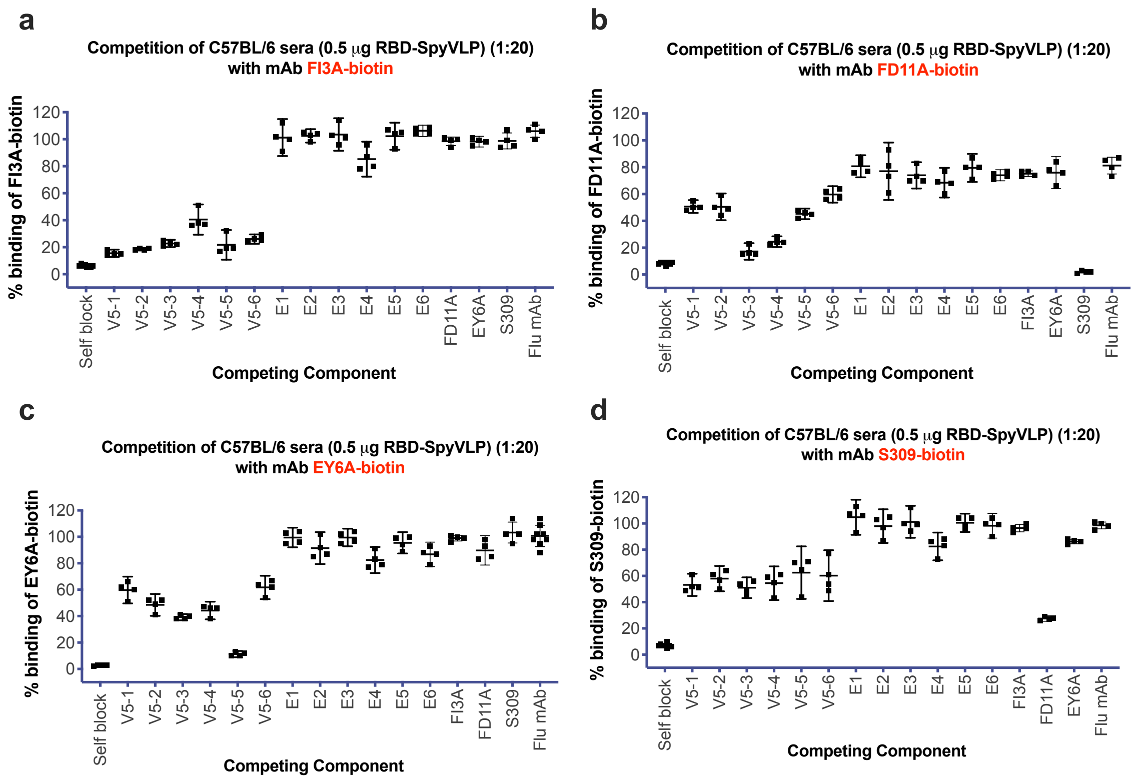

Figure S6: Competition ELISA of C57BL/6 sera with RBD mAbs binding to non-overlapping epitopes on RBD. Competition ELISA of C57BL/6 mice immunised with 0.5 µg RBD-SpyVLP (sera from 3 weeks post boost) against (a) FI-3A biotin, (b) FD11A biotin (competes with S309), (c) EY6A biotin and (d) S309 biotin. 1:20 serum dilutions were used and each serum was tested in quadruplicate. Data are shown as the mean of the readings ± 95% confidence interval. Self block= the same mAb (non-biotinylated) was used to compete with the biotinylated equivalent. Flu mAb= a mAb targeting the influenza haemagglutinin stem. V5-1 to V5-6= sera from mice immunised with 0.5 µg RBD-SpyVLP. E1 to E6= sera from mice immunised with empty VLP.

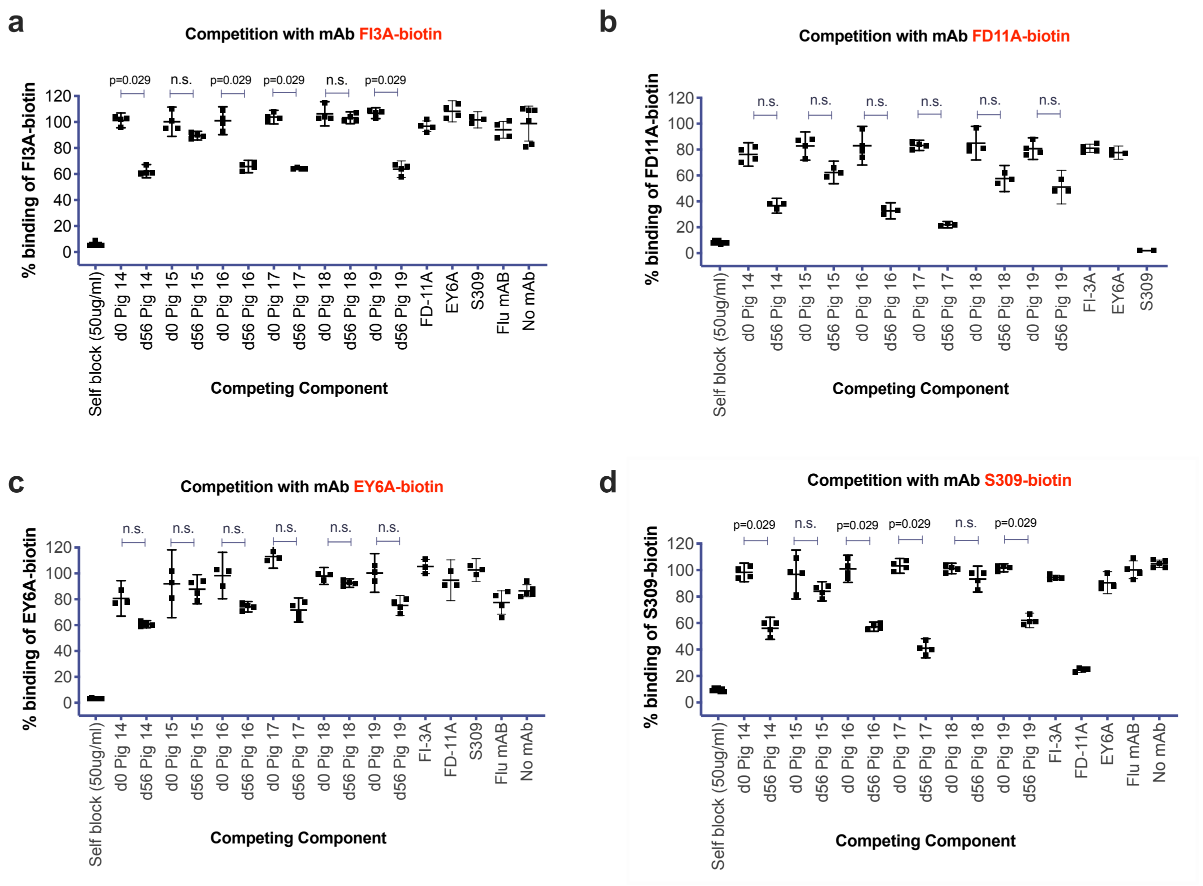

Figure S7: Competition ELISA of pig sera with RBD mAbs binding to non-overlapping epitopes on RBD. (A) Competition ELISA of preimmune sera (day 0) and post boost sera (day 56) for pigs (n=3) immunised with 5 µg RBD-SpyVLP (pig 14, 15 & 16) or 50 µg RBD-SpyVLP (pig 17, 18 & 19) against (A) FI-3A biotin, (B) FD11A biotin, (C) EY6A biotin and (D) S309 biotin. Data are shown as the mean of the three or four readings ± 95% confidence interval. Statistical analysis between pre-immunised (day 0) and day 56 sera was analysed using two-tailed Mann Whitney U. n.s = not significant. Self block= the same mAb (non-biotinylated) was used to compete with the biotinylated equivalent. Flu mAb= a mAb targeting the influenza haemagglutinin stem.

VNT on pooled sera (C57BL/6)		
**Group**	**Probit mid-point $PRNT_{50}$ dilution (ND_{50})**	**95% confidence intervals**
**0.1 µg RBD**	395	113-751
**0.5 µg RBD**	79	16-159
**0.1 µg RBD-SpyVLP**	6,087	4,487-9,375
**0.5 µg RBD-SpyVLP**	8,789	6,710-12,966
**VLP**	122	89-147
**MERS control sera**	527	419-651

Table S1: VNT of pooled sera from C57BL/6 mouse immunisation. The table summarises the ND_{50} of pooled sera of C57BL/6 mice (n=6) immunised with RBD, RBD-SpyVLP or VLP against live SARS-CoV-2 in a plaque reduction-based neutralisation assay. A serum panel from humans infected with MERS-CoV, known to cross-neutralise SARS-CoV-2 (National Institute for Biological Standards and Control, UK), was included as an assay control.

RBD-SpyVLP	
**Data collection and reconstruction**	
Voltage (kV)	300
Frames	40
Dose rate (e-/ Å^2/ s)	8.6
Total dose (e-/ Å^2)	48.1
Pixel size (Å)	0.815
Defocus Range (µm)	0.8 - 2.6
Symmetry	I1 [C1]
Movies	7,393
Particles in final reconstruction	156,785
Refined map resolution (Å)	3.7 [4.9]
FSC threshold	0.143
Map sharpening B-factor (Å^2)	206.9 [155.3]

Table S2: Cryo-EM data collection parameters.

Cryo-EM data collection, refinement and validation statistics

	RBD-SpyVLP (EMDB-11997) (PDB 7B3Y)
**Data collection and processing**	
Magnification	165 kX
Voltage (kV)	300
Electron exposure (e^{-}/Å^2)	48.1
Defocus range (μm)	-0.8 to -2.6
Pixel size (Å)	0.815
Symmetry imposed	I1
Movies collected (no.)	7393
Initial particles after 2D classification (no.)	187,042
Final particles used in reconstruction (no.)	150,785
Map resolution (Å)	3.7
FSC threshold	0.143
Map resolution range (Å)	3 - 9
**Rigid body Refinement without RBD-Spy**	
Initial model used (PDB code)	5kp9
Model resolution (Å)	3.8/4.3
FSC threshold	0.143/0.5
Map sharpening *B* factor (Å^2)	206.9
Model composition	
Non-hydrogen atoms	1516
Protein residues	202
Ligands	0
B factors (Å^2)	
Protein	9.4
Ligand	0
R.m.s. deviations	
Bond lengths (Å)	0.014
Bond angles (°)	1.44
Validation	
MolProbity score	1.7
Clashscore	4.5
Poor rotamers (%)	4.4
Ramachandran plot	
Favored (%)	98.0
Allowed (%)	2.0
Disallowed (%)	0.0

Table S3: Cryo-EM data collection, confinement and validation statistics.

1. Walls, A.C. et al. Structure, Function, and Antigenicity of the SARS-CoV-2 Spike Glycoprotein. *Cell* **181**, 281-292 e286 (2020).
