# Supplementary material for: A COVID-19 vaccine candidate using SpyCatcher multimerization of the SARS-CoV-2 spike protein receptor-binding domain induces potent neutralising antibody responses
Source: Nat Commun. 2021 Jan 22;12:542. doi: 10.1038/s41467-020-20654-7 (PMC7822889; doi:10.1038/s41467-020-20654-7)
Supplement: Supplementary file 2 — Reporting Summary [file 41467_2020_20654_MOESM2_ESM.pdf]

nature research

Corresponding author(s): Alain Townsend, Tiong Kit Tan, Mark Howarth

Last updated by author(s): 03-12-20

Reporting Summary

Nature Research wishes to improve the reproducibility of the work that we publish. This form provides structure for consistency and transparency in reporting. For further information on Nature Research policies, see our Editorial Policies and the Editorial Policy Checklist.

Statistics

For all statistical analyses, confirm that the following items are present in the figure legend, table legend, main text, or Methods section.

n/a	Confirmed	
☐	☒	The exact sample size (*n*) for each experimental group/condition, given as a discrete number and unit of measurement
☐	☒	A statement on whether measurements were taken from distinct samples or whether the same sample was measured repeatedly
☐	☒	The statistical test(s) used AND whether they are one- or two-sided<br>*Only common tests should be described solely by name; describe more complex techniques in the Methods section.*
☒	☐	A description of all covariates tested
☐	☒	A description of any assumptions or corrections, such as tests of normality and adjustment for multiple comparisons
☐	☒	A full description of the statistical parameters including central tendency (e.g. means) or other basic estimates (e.g. regression coefficient) AND variation (e.g. standard deviation) or associated estimates of uncertainty (e.g. confidence intervals)
☐	☒	For null hypothesis testing, the test statistic (e.g. F, t, r) with confidence intervals, effect sizes, degrees of freedom and P value noted<br>*Give P values as exact values whenever suitable.*
☒	☐	For Bayesian analysis, information on the choice of priors and Markov chain Monte Carlo settings
☒	☐	For hierarchical and complex designs, identification of the appropriate level for tests and full reporting of outcomes
☐	☒	Estimates of effect sizes (e.g. Cohen's *d*, Pearson's *r*), indicating how they were calculated

Our web collection on statistics for biologists contains articles on many of the points above.

Software and code

Policy information about availability of computer code

Data collection	No software was used in data collection
Data analysis	GraphPad Prism 8<br>LabView2020<br>ChimeraX<br>SerialEM3.8.0 beta

For manuscripts utilizing custom algorithms or software that are central to the research but not yet described in published literature, software must be made available to editors and reviewers. We strongly encourage code deposition in a community repository (e.g. GitHub). See the Nature Research guidelines for submitting code & software for further information.

Data

Policy information about availability of data

All manuscripts must include a data availability statement. This statement should provide the following information, where applicable:
- Accession codes, unique identifiers, or web links for publicly available datasets
- A list of figures that have associated raw data
- A description of any restrictions on data availability

The datasets generated during and/or analysed during the current study are available from the corresponding author on reasonable request.
SpyTag-RBD GenBank MT945427 and SpyCatcher003-mi3 GenBank MT945417.

Field-specific reporting

Please select the one below that is the best fit for your research. If you are not sure, read the appropriate sections before making your selection.

- [x] Life sciences
- [ ] Behavioural & social sciences
- [ ] Ecological, evolutionary & environmental sciences

For a reference copy of the document with all sections, see nature.com/documents/nr-reporting-summary-flat.pdf

Life sciences study design

All studies must disclose on these points even when the disclosure is negative.

Sample size	Power analysis from the first mouse experiment showed that n=6 is giving at least 80% power. Therefore 6 mice per group was used in subsequent experiments. No statistical methods were used to determine sample size for the in vitro assays. However, at least 3 independent biological replicates were measured and the each samples was measure in at least duplicates in each assays. We titrate the samples whenever possible to assess dose-activity relationship and repeat experiments whenever possible to assess assay variation. Sample size was chosen based on prior knowledge in the respective assays and their intrinsic variability
Data exclusions	No data was excluded.
Replication	Independent mouse experiments have been repeated in this study with the same dose of the RBD-SpyVLP vaccine and the immunogenicity results were reproducible. The mouse experiment with the RBD-SpyVLP has been replicated successfully multiple times to date. For instance, Group 0.5ug RBD-SpyVLP in Figure 2b and group "pre-lyophilisation" in Figure 2E are 2 independent experiments performed in the same species of mice with the same vaccine regimen. The other independent repeats were successful and show similar levels of immune responses.
Randomization	Allocation of samples was random
Blinding	The immunisation and analysis of mice experiments were not blinded as the experiments were carried out during the early lockdown in the UK therefore the experiments were performed by a single person. However, the neutralisation assays carried out at The Crick Institute and Public Health England on the mouse sera were blinded. For the pig experiments, the dosing and analysis were done by different groups of people but samples were not coded therefore it was not fully blinded. The CPE for the microneutralisation assays was analysed by pathologists blinded to the identity of the samples.

Reporting for specific materials, systems and methods

We require information from authors about some types of materials, experimental systems and methods used in many studies. Here, indicate whether each material, system or method listed is relevant to your study. If you are not sure if a list item applies to your research, read the appropriate section before selecting a response.

Materials & experimental systems

n/a	Involved in the study	
[ ]	[x]	Antibodies
[ ]	[x]	Eukaryotic cell lines
[x]	[ ]	Palaeontology and archaeology
[ ]	[x]	Animals and other organisms
[ ]	[x]	Human research participants
[x]	[ ]	Clinical data
[x]	[ ]	Dual use research of concern

Methods

n/a	Involved in the study	
[x]	[ ]	ChIP-seq
[ ]	[x]	Flow cytometry
[x]	[ ]	MRI-based neuroimaging

Antibodies

Antibodies used

CR3022, VHH72-Fc, S309 were produced in house based on published sequences as stated in the main text.
FD-11A, FD-5D, FN-12A, FM-7B, ,FI-3A EY-6A, FI-4A and FI-1C were novel antibodies isolated in our lab as described in KA Huang et al. bioRxiv 2020 https://doi.org/10.1101/2020.08.28.267526.

HRP-goat-anti-human IgG antibody (Dako, P0214)
HRP goat anti-mouse antibody (Dako P0447)
Alexa Fluor 647 goat anti-human antibody (Life Technologies A21455)
rabbit polyclonal anti-NSP8 antibody (Antibodies Online; ABIN233792)
Goat anti-rabbit-HRP conjugate (Bio-Rad, 1706515)

CD3-FITC mAb (clone BB23-8E6-8C8, BD Biosciences 559582)
CD4-PerCP-Cy5.5 mAb (clone 74-12-4, BD Bioscience 561474)

CD8α-PE mAb (clone 76-2-11, BD Bioscience 559584)
IFN-γ-Alexa Fluor 647 mAb (clone CC302, Bio-Rad MCA1783)
TNF-α-Brilliant Violet 421 mAb (clone Mab11, BioLegend 502931)
streptavidin-Brilliant Violet 421 (BioLegend 405226)
streptavidin-Brilliant Violet 650 (BioLegend 405231)
streptavidin-PerCP Cy5.5 (BioLegend 405214)
Zombie Aqua, CD3-PE-Cy7 mAb (clone BB23-8E6-8C8, BD Biosciences 561477)
CD14-PE Vio 770 mAb (clone REA599, Miltenyi Biotec 130-110-520)
Pig IgG-Alexa Fluor-647 mAb (CSA 3841, Cohesion Biosciences, Generon)
Goat anti-pig IgA-FITC polyclonal Ab (BioRad AAI40)
Mouse anti-pig IgM-PE mAb (clone K52 1C3, BioRad MCA637GA)
Mouse anti-porcine IgG mAb (clone MT421, Mabtech, 2BScientific)
biotinylated anti-IgG mAb (clone MT424-biotin, Mabtech 3151-3-250)
streptavidin-alkaline phosphatase (streptavidin-ALP) enzyme conjugate (Mabtech 3310-10-1000)
Goat Anti-Pig IgG HRP (Abcam, ab6915, polyclonal)
Goat anti-porcine IgA HRP (Bio-Rad Antibodies, AAI40P, polyclonal)
Goat anti-porcine IgG HRP (Bio-Rad Antibodies, AAI41P, polyclonal)

Validation

Human monoclonal antibodies to RBD protein are validated in following studies: ter Meulen et al 2006 Plos Med, Wrapp et al 2020 Cell, Zhou et al 2020 Nat Struct Mol Biol, Pinto et al 2020 Nature and Huang et al 2020 bioxRiv Manuscript submitted to Nature Communications.

HRP-goat-anti-mouse IgG antibody (Dako, P0447): Intended for use in immunohisochemistry, immunoblotting, and ELISA. The cross-reaction with human immunoglobulins and fetal calf serum is negligible. The HRP-conjugated antibody reacts with all mouse IgG subclasses and mouse IgM, and it will probably also react with other mouse immunoglobulins via their light chains. Cross-reaction with human immunoglobulins and fetal calf serum has been removed by solid-phase absorption and absorbed specific antibody has been isolated by affinity chromatography on a column with mouse immunoglobulins. However, it should be noted that the cross-reaction with rabbit immunoglobulins is very low. The goat antibody used for horseradish peroxidase-conjugation may cross-react with immunoglobulins from other species.

HRP rabbit anti-human antibody (Dako P0214): The antibody reacts with the gamma-chains of human IgG. Traces of contaminating antibodies have been removed by solid-phase absorption. Before conjugation the specificity of the antibody has been ascertained as follows: Crossed immunoelectrophoresis: Only the IgG immunoprecipitation arch appears when the antibody is tested against human plasma. Staining: Coomassie Brilliant Blue. ELISA: No significant reaction is seen in indirect ELISA when using human IgA and IgM as coating antigens. In double antibody sandwich ELISA no significant reaction is observed with human plasma stripped of IgG. Cross-reaction with IgG from other species may occur. As demonstrated by rocket immunoelectrophoresis, the unconjugated antibody cross-reacts with the IgG-equivalent protein in cat, cow, deer, dog, goat, horse, mink, monkey, mouse, musk ox, polecat, raccoon dog, rat and swine (6.)

Alexa Fluor 647 goat anti-human antibody (Life Technologies A21455) : To minimize cross-reactivity, these goat anti-human IgG (H+L) whole secondary antibodies have been affinity purified and cross-adsorbed against mouse, rabbit, and bovine serum prior to conjugation. Cross-adsorption or pre-adsorption is a purification step to increase specificity of the antibody resulting in higher sensitivity and less background staining. The secondary antibody solution is passed through a column matrix containing immobilized serum proteins from potentially cross-reactive species. Only the nonspecific-binding secondary antibodies are captured in the column, and the highly specific secondaries flow through. The benefits of this extra step are apparent in multiplexing/multicolor-staining experiments (e.g., flow cytometry) where there is potential cross-reactivity with other primary antibodies or in tissue/cell fluorescent staining experiments where there are may be the presence of endogenous immunoglobulins.

Rabbit polyclonal anti-NSP8 antibody (Antibodies Online; ABIN233792): This whole rabbit serum was produced by repeated immunizations with a purified His- tagged recombinant protein corresponding to full-length SARS-Coronavirus nsp8. Immunogentype:Recombinant. This antibody has been tested for use in immunofluorescence microscopy, immunoelectron microscopy, immunoprecipitation and by western blot. Specific conditions for reactivity should be optimized by the end user. Expect a band of approximately 22 kDa in size corresponding to SARS-CoV nsp8 by western blotting in the appropriate cell lysate or extract. For immunofluorescence microscopy, Vero-E6 cells, grown on glass slides, were infected with SARS-CoV-Fr1 strain for 1 h at 37°C. Infection occurred in PBS/DEAE/2%FCS followed by exchange to EMEM/25mMHEPES/2%FCS. Cells were fixed with PBS/3%PFA. After washing fixed cells, antibody incubation was performed in PBS/5%FCS for 30 min.

Goat anti-rabbit-HRP conjugate (Bio-Rad, 1706515): Is prepared from antisera raised in goats immunised with purified rabbit IgG. Immunoaffinity chromatography procedures are used to isolate antibodies and to remove antibodies which cross-react with human immunoglobulin.

CD3-FITC mAb (clone BB23-8E6-8C8, BD Biosciences 559582) : The BB23-8E6-8C8 monoclonal antibody specifically binds to the 25-kDa ε chain of the T-cell receptor-associated CD3 complex. It recognizes all CD4+ and most CD8+ peripheral blood T lymphocytes, most thymocytes and phytohemagglutinin-stimulated blasts, and subsets of spleen and Peyer's patch lymphocytes. BB23-8E6-8C8 is a immunoglobulin isotype switch variant of the BB23-8E6 clone. This isotype-switch variant induces a proliferative response of peripheral blood mononuclear cells. The epitope recognized by BB23-8E6 mAb was designated CD3a by the Second International Swine CD Workshop. Isotype: Mouse (BALB/c) IgG2b, κ. Reactivity: QC Testing: Pig

CD4-PerCP-Cy5.5 mAb (clone 74-12-4, BD Bioscience 561474): The 74-12-4 (also known as clone PT4) monoclonal antibody

specifically binds to CD4, a 55-kDa antigen expressed on T lymphocytes. This antibody does not react with CTL effectors, CTL precursors, or NK cells (ie, CD8[bright] cells) and it does not cross-react with human or bovine cells. Two peripheral T-helper lymphocyte phenotypes can be distinguished in the pig: CD4+CD8- and CD4+CD8[dull]. mAb 74-12-4 has been reported to inhibit proliferative responses of peripheral blood lymphocytes to mitogen, soluble antigen, and alloantigen. It is only marginally effective for in vivo depletion of peripheral CD4+ T cells. Two alloantigenic forms of CD4 have been recognized in miniature swine based upon their recognition (CD4.1) or lack of recognition (CD4.2) by mAb 74-12-4; the CD4.2 phenotype displays an autosomal recessive, non-MHC-linked, pattern of inheritance. The molecular basis for the polymorphism is a cluster of nucleotide differences leading to multiple amino-acid substitutions in the Ig CDR2-like loop structure. This mAb was clustered as anti-CD4a at the First International Swine CD Workshop. It has been reported to crossreact with chicken leukocytes. Isotype: Mouse (BALB/c) IgG2b, κ. Reactivity: QC Testing: Pig

CD8α-PE mAb (clone 76-2-11, BD Bioscience 559584): The 76-2-11 (also known as clone PT8) antibody reacts with an epitope on the CD8α chain, a 35-kDa antigen expressed on thymocytes, peripheral T lymphocytes, and NK cells. The CD8 molecule can exist as a 70 kDa homodimer, composed of α chains, or heterodimer, composed of an α and β chain. Cells which express the CD8αα homodimer display dimmer staining with mAb 76-2-11 than CDαβ-expressing cells. The 76-2-11 mAb does not cross-react with human or bovine cells. Two peripheral CD8+ T-cell populations can be distinguished in the pig: CD8-bright CD4- CTL effectors/precursors and CD8-dull CD4+ T-helper lymphocytes. Pig NK cells express CD8 (dull staining), CD2, MHC class II, LFA-1, and asialo-GM1, but not CD3, CD4, CD5, or CD6. mAb 76-2-11 has been reported to partially inhibit in vitro cytotoxic activity of PBL to allogeneic leukocytes, but not NK-cell-mediated lysis, and to deplete CD8+ T cells in vivo. This clone was clustered as anti-CD8a at the First International Swine CD workshop. . Isotype: Mouse (BALB/c) IgG2b, κ. Reactivity: QC Testing: Porcine

IFN-γ-Alexa Fluor 647 mAb (clone CC302, Bio-Rad MCA 1783): recognizes bovine interferon-gamma, a 143 amino acid cytokine with potent activating, antiviral and anti proliferitive properties, produced as a pro-peptide with an additional 23 amino acid N-terminal signal peptide sequence having a molecular weight of ~20 kDa. IFNγ is predominantly secreted by activated T lymphocytes in response to specific mitogens as a result of infection (Rhodes et al. 2000). Mouse anti bovine γ inerferon antibody, clone CC302 has been demonstrated to be reactive to a number of mammalian species including human, sheep, dog, pig, goat and mink (Pedersen et al. 2002). Clone CC302 has been successfully used for the evaluation of γ interferon levels in the sera of calves naturally infected with M. avium. subsp paratuberculosis (Appana et al. 2013) as a detection reagent using an ELISA.

TNF-α-Brilliant Violet 421 mAb (clone Mab11, BioLegend 502931): Reactivity - Human, Cat (Feline) Cross-Reactivity: Chimpanzee, Baboon, Cynomolgus, Rhesus, Pigtailed Macaque, Sooty Mangabey, Swine(Pig, Porcine). Each lot of this antibody is quality control tested by intracellular immunofluorescent staining with flow cytometric analysis.

streptavidin-Brilliant Violet 421 (BioLegend 405226): Reactivity: Human, Mouse, Rat, All Species. Each lot is quality control tested by immunofluorescent staining with flow cytometric analysis.

streptavidin-Brilliant Violet 650 (BioLegend 405231): Human, Mouse, Rat, All Species. Each lot is quality control tested by immunofluorescent staining with flow cytometric analysis.

streptavidin-PerCP Cy5.5 (BioLegend 405214): Human, Mouse, Rat, All Species. Each lot is quality control tested by immunofluorescent staining with flow cytometric analysis.

Zombie Aqua, CD3-PE-Cy7 mAb (clone BB23-8E6-8C8, BD Biosciences 561477): The BB23-8E6-8C8 monoclonal antibody specifically binds to the 25-kDa ε chain of the T-cell receptor-associated CD3 complex. It recognizes all CD4+ and most CD8+ peripheral blood T lymphocytes, most thymocytes and phytohemagglutinin-stimulated blasts, and subsets of spleen and Peyer's patch lymphocytes. BB23-8E6-8C8 is a immunoglobulin isotype switch variant of the BB23-8E6 clone. This isotype-switch variant induces a proliferative response of peripheral blood mononuclear cells. The epitope recognized by BB23-8E6 mAb was designated CD3a by the Second International Swine CD Workshop. Isotype: Mouse (BALB/c) IgG2b, κ. Reactivity: QC Testing: Porcine.

CD14-PE Vio 770 mAb (clone REA599, Miltenyi Biotec 130-110-520): Clone REA599 recognizes the human CD14 antigen. CD14 is part of the functional heteromeric LPS receptor complex comprised of at least CD14, TLR4, and MD-2. It up-regulates cell surface molecules, including adhesion molecules. CD14 is strongly expressed on most human monocytes and macrophages in peripheral blood, other bodily fluids, and in various tissues such as lymph nodes and spleen. CD14 is weakly expressed on subpopulations of human neutrophils and myeloid dendritic cells.
Additional information: Clone REA599 displays negligible binding to Fc receptors. QC tested. Species reactivity: human. Crossreactivity: cynomolgus monkey (Macaca fascicularis), dog, goat, cat, rabbit, mink, bovine, pig, sheep.

Pig IgG-Alexa Fluor-647 mAb (CSA3841, Cohesion Biosciences, Generon): By immunoelectrophoresis and ELISA this antibody reacts specifically with Pig IgG. No antibody was detected against non immunoglobulin serum proteins. Species reactivity key: H- Human, M-Mouse, R- Rat, B- Bovine, C- Chicken, D- Dog, G- Goat, Mk- Monkey, P- Pig, Rb- Rabbit, S- Sheep, Z- Zebrafish

Goat anti-pig IgA-FITC polyclonal Ab (BioRad AAI40): recognizes porcine IgA and shows no cross-reactivity with other porcine immunoglobulin classes as assessed by immunoelectrophoresis. This antibody may cross-react with IgA from other species. Goat anti Porcine IgA antibody has been succesfully used for the evaluation of porcine IgA levels in body fluids of pigs by both ELISA and Western blotting.

Mouse anti-pig IgM-PE mAb (clone K52 1C3, BioRad MCA637GA): recognizes porcine IgM heavy chain. No cross-reactivity with porcine IgA and IgG is seen in ELISA.

Mouse anti-porcine IgG mAb (clone MT421, Mabtech, 2BScientific): Specificity porcine IgG. Cross-reacts with IgG from horse (30% cross-reactivity) and goat (5%).

biotinylated anti-IgG mAb (clone MT424-biotin, Mabtech 3151-3-250) : Specificity: Native porcine IgG. No cross-reactivity (<0.1%) with IgG from cow, horse, sheep, goat, human and rat. This monoclonal antibody enables sensitive and specific detection of porcine IgG in immunoassays such as ELISA and ELISpot.

Streptavidin-alkaline phosphatase (streptavidin-ALP) enzyme conjugate (Mabtech 3310-10-1000): Highly purified enzyme from calf-intestine with high activity especially suited for enzyme immunoassay purposes. The preparation has been thoroughly depleted of free ligand and free enzyme. Optimised for ELISpot.

CD14-PE Vio 770 (Miltenyi) Clone REA599 recognizes the human CD14 antigen. CD14 is part of the functional heteromeric LPS receptor complex comprised of at least CD14, TLR4, and MD-2. It up-regulates cell surface molecules, including adhesion molecules. CD14 is strongly expressed on most human monocytes and macrophages in peripheral blood, other bodily fluids, and in various tissues such as lymph nodes and spleen. CD14 is weakly expressed on subpopulations of human neutrophils and myeloid dendritic cells. Additional information: Clone REA599 displays negligible binding to Fc receptors. Cross reactivity: cynomolgus monkey (Macaca fascicularis), dog, goat, cat, rabbit, mink, bovine, pig, sheep

Mouse anti-porcine IgG (clone MT421, Mabtech, 2BScientific). Specific to porcine IgG, cross-reacts with IgG from horse (30% cross-reactivity) and goat (5% cross-reactivity).

Eukaryotic cell lines

Policy information about cell lines

Cell line source(s)	MDCK-SIAT1 (ECACC 05071502), MDCK-RBD (produced in house using lentiviral transduction), MDCK-Spike (produced in house using lentiviral transduction), Expi293F (ThermoFisherScientific), Vero-E6 (ECACC)
Authentication	None of the cell line produced in house was authenticated. The Expi293F cell line was authenticated by ThermoFisherScientific. Vero-E6 was authenticated by ECACC
Mycoplasma contamination	The cell lines were tested to be free of mycoplasma
Commonly misidentified lines (See ICLAC register)	no commonly misidentified cell lines were used in the study

Animals and other organisms

Policy information about studies involving animals; ARRIVE guidelines recommended for reporting animal research

Laboratory animals	Mouse (C57BL/6 and BALB/c), female, aged 5-6 weeks at the time of immunisation.
Wild animals	Large White-Landrace-Hampshire cross-bred pigs of 8–10 weeks of age from a UK commercial rearing unit. Pigs were humanely euthanised using overdose of anaesthetic via intravenous route at the end of the study
Field-collected samples	The study did not involve field-collected samples
Ethics oversight	University of Oxford (mouse) and The Pirbright Institute (pig) Local Ethical Review Body

Note that full information on the approval of the study protocol must also be provided in the manuscript.

Human research participants

Policy information about studies involving human research participants

Population characteristics	Patients were recruited at the John Radcliffe Hospital between March to May 2020. All patients were confirmed to have a test positive for SARS-CoV-2 using reverse transcriptase polymerase chain reaction (RT-PCR) from an upper respiratory tract (nose/throat) swab tested in accredited laboratories. The degree of severity was identified as mild, severe or critical infection according to recommendations from the World Health Organisation as described in Peng et al 2020 (https://doi.org/10.1038/s41590-020-0782-6).
Recruitment	Convalescent plasma samples (18 mild, 10 severe/critical) were randomly selected from three ethically approved COVID19 cohorts recruited at John Radcliffe Hospital between March and May 2020. The degree of severity was identified as mild,

severe or critical infection according to recommendations from the World Health Organisation. Time between onset of symptoms and sampling were known for all patients and if labelled as convalescent patients were sampled at least 28 days from the start of their symptoms. Written informed consent was obtained from all patients as described in Peng et al 2020

Ethics oversight

Gastro-intestinal illness in Oxford: COVID substudy [Sheffield Research Ethics Committee, reference: 16/YH/0247]ISARIC/WHO, Clinical Characterisation Protocol for Severe Emerging Infections [Oxford Research Ethics Committee C, reference 13/SC/0149], the Sepsis Immunomics project [Oxford Research Ethics Committee, reference:19/SC/0296]) and by the Scotland A Research Ethics Committee (Ref: 20/SS/0028).

Note that full information on the approval of the study protocol must also be provided in the manuscript.

Flow Cytometry

Plots

Confirm that:

- [x] The axis labels state the marker and fluorochrome used (e.g. CD4-FITC).
- [x] The axis scales are clearly visible. Include numbers along axes only for bottom left plot of group (a 'group' is an analysis of identical markers).
- [x] All plots are contour plots with outliers or pseudocolor plots.
- [x] A numerical value for number of cells or percentage (with statistics) is provided.

Methodology

Sample preparation

Whole blood (mouse) was collected from bleeding through the tail vein or via cardiac puncture. Whole blood was allowed to clot at RT for 30min to 1 hour and spun down to collect sera. Sera was heat inactivated at 56C for 30min and store at 4C prior to assays. Sera was stored at -20C for long term storage.

Whole blood was taken by venepuncture of the external jugular vein: 8 mL/pig in BD SST vacutainer tubes (Fisher Scientific) for serum collection. Sera samples were stored at -20 °C and heat-inactivated at 56 °C for 2 h prior to assays. Oral and nasal swabs were collected weekly and placed in 500 µL Media 199 (Thermo Fisher) supplemented with 0.0025% Nystatin (Merck), 0.01% Penicillin-Streptomycin (Gibco), 0.025% 1M HEPES solution (Gibco), 0.005% (w/v) sodium bicarbonate (Merck) and 0.067% (w/v) BSA (Merck) (VTM). Swabs were centrifuged at 700 × g for 5 min before aspirating the liquid and storing with the swab at -20 °C. Prior to assessment of antibodies, swabs and VTM were loaded in Spin-X Centrifuge 0.45 µm columns (Fisher Scientific) and fluid collected by centrifugation at 21,000 × g for 5 min.
For PBMC isolation, whole blood was taken by venepuncture of the external jugular vein: 40 mL/pig in BD heparin vacutainer tubes (Fisher Scientific). PBMCs were isolated from heparinized blood by density gradient centrifugation and suspended at 1 × 10^7 cells/mL in RPMI-1640 medium, GlutaMAX supplement, HEPES (Gibco) supplemented with 10% (v/v) heat-inactivated FBS (New Zealand origin, Life Science Production), 1% Penicillin-Streptomycin and 0.1% 2-mercaptoethanol (50 mM; Gibco) (cRPMI).

Instrument

ChemiDoc XRS imager (Bio-Rad)
BenchTop 2K freeze-dryer (VirTis)
Clariostar Plus plate reader (BMG Labtech)
Omnisizer (Victotek)
Titan Krios G2 (Thermo Fischer)
GloMax-Multi+ Detection System (Promega)
LSRFortessa (BD)
CTL ImmunoSpot Analyzer (Cellular Technologies)

Software

ImageLab (version 5.2) software (Bio-Rad)
Fiji distribution of ImageJ (version 1.51n)
SerialEM
cryoSPARC (v.2.14.1)
Chimera
LabView

Cell population abundance

An acquisition threshold was set at a minimum of 100,000 events in the live gate for T cell analysis and 200,000 events in the live gate for B cell analysis.

Gating strategy

Antigen-specific T cells were identified by gating on LIVE/DEAD negative, doublet negative (FSC-H vs FSC-A), size (FSC-H vs SSC), CD3+CD4+ or CD3+CD4-CD8+ cells and cytokine positive. Cytokine positive responses are presented after subtraction of the background response detected in the corresponding unstimulated sample (media and Golgi-plug) of each individual

PBMC sample.
Antigen-specific B cells were identified by first gating on LIVE/DEAD negative, doublet negative (FSC-H vs FSC-A), size (FSC-H vs SSC), CD3-CD14-IgM+, CD3-CD14-IgA+ or CD3-CD14-IgG+ cells. Following exclusion of cells binding to NiV G decoy tetramer, cells were gated on dual staining with SARS-CoV-2 S RBD tetramers conjugated to BV421 and BV650.

☒ Tick this box to confirm that a figure exemplifying the gating strategy is provided in the Supplementary Information.
